# Vice-versa: The iron trade in the western Roman Empire between Gaul and the Mediterranean

**DOI:** 10.1371/journal.pone.0268209

**Published:** 2022-05-17

**Authors:** Gaspard Pagès, Philippe Dillmann, Enrique Vega, Marion Berranger, Sylvain Bauvais, Luc Long, Philippe Fluzin

**Affiliations:** 1 CNRS, Archéologies et Sciences de l’Antiquité, ArScAn, UMR7041, Équipe Archéologie de la Gaule et du Monde Antique (GAMA), MSH Mondes, 21 allée de l’université, 92023, Nanterre cedex, France; 2 CNRS, LAPA-IRAMAT, NIMBE, CEA, CNRS, Université Paris-Saclay, CEA Saclay 91191, Gif-sur-Yvette, France; 3 CNRS, LMC-IRAMAT, UMR7065, Université de Technologie Belfort Montbéliard, CNRS, Montbéliard, France; 4 Département des Recherches Archéologiques Subaquatiques et Sous-Marines (DRASSM), Archéologie des Sociétés Méditerranéennes, ASM UMR 5140, 34000, Montpellier, France; Ghent University, BELGIUM

## Abstract

Starting from the second century BC, with the fast expansion of the Roman Empire, iron production and consumption developed exponentially in north-western Europe. This rapid growth naturally led to an increase in trade, that still remains to be studied encompassing a broad scope, so as to not neglect long-distance exchanges. This is today possible by taking advantage of the progress made in the past 40 years in archaeology and archaeometallurgy. Cargoes of iron bars recovered from a group of 23 wrecks located off the coast of Saintes-Maries-de-la-Mer (Bouches-du-Rhône, France), opposite an old branch of the Rhône River, constitute a rich opportunity to examine this trade, by comparing the slag inclusions trapped in iron bars to primary slag from the six main ironmaking areas in Gaul. Based on a trace element analysis of these inclusions and this slag, we suggest that ships travelled down the Rhône carrying iron produced in Wallonia (Belgium), while others sailed up the Rhône transporting iron produced in Montagne Noire (Aude, France).

## 1.Introduction

Trade in foodstuffs (e.g., wine, brine, smoked meat) and in heavy raw materials (both metallic and non-metallic, such as copper, lead, marble) during the Roman Empire followed complex traffic patterns. Trading routes crisscrossed and intertwined around the Mediterranean, sometimes anchored in older channels established during the Republic period [1]. This article deals with this aspect of research with a special focus on south-to-north and north-to-south flows in the iron trade, between the Mediterranean and Gaul.

The exploitation of mineral resources in the territories conquered by Rome since the Republic period was of prime importance. It generated large-scale trade to supply all spheres of expanding Roman civilization with raw and semi-finished iron products. From the 2nd century BC onwards, iron became a widespread material used in all aspects of Gallic and Roman life–household activities, arts and crafts, weapon-making, construction and all forms of architecture [2]. Maritime trade played a major role in this trend because it allowed, as it continues to do today, the circulation of heavy materials over great distances, and because the Mediterranean Sea was the heart of the Roman Empire, as indicated by its Latin name: *Mare nostrum* (“Our Sea”).

**Fig 1:**
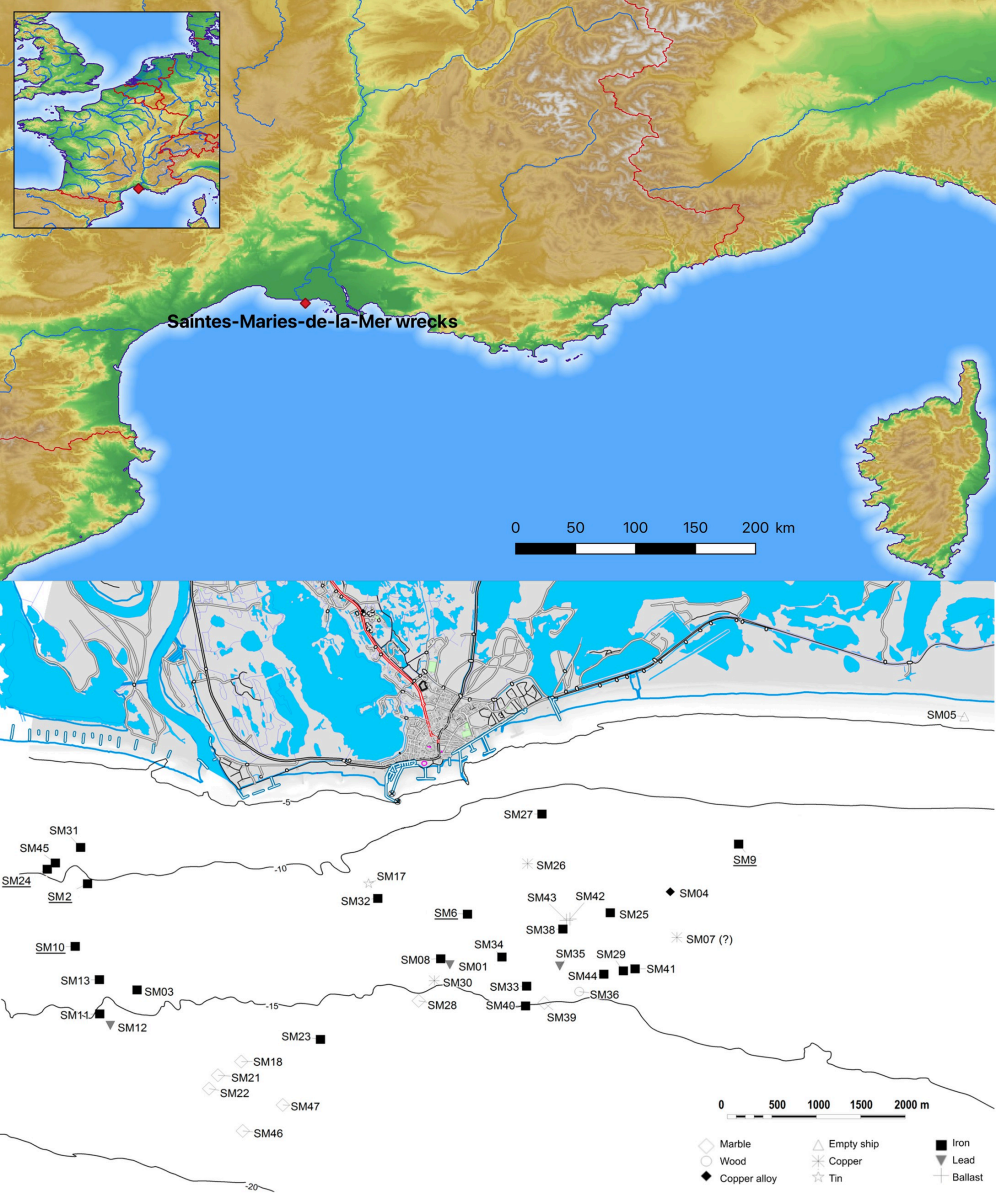
**Antique wrecks (named SM with an ID number) off the coast of Saintes-Maries-de-la-Mer (Bouches-du-Rhône, France; DEM NASA Shuttle Radar Topography Mission Global 1 arc second) and types of cargo (north at the top).** The iron bars studied come from the underlined wreck names.

**Fig 2:**
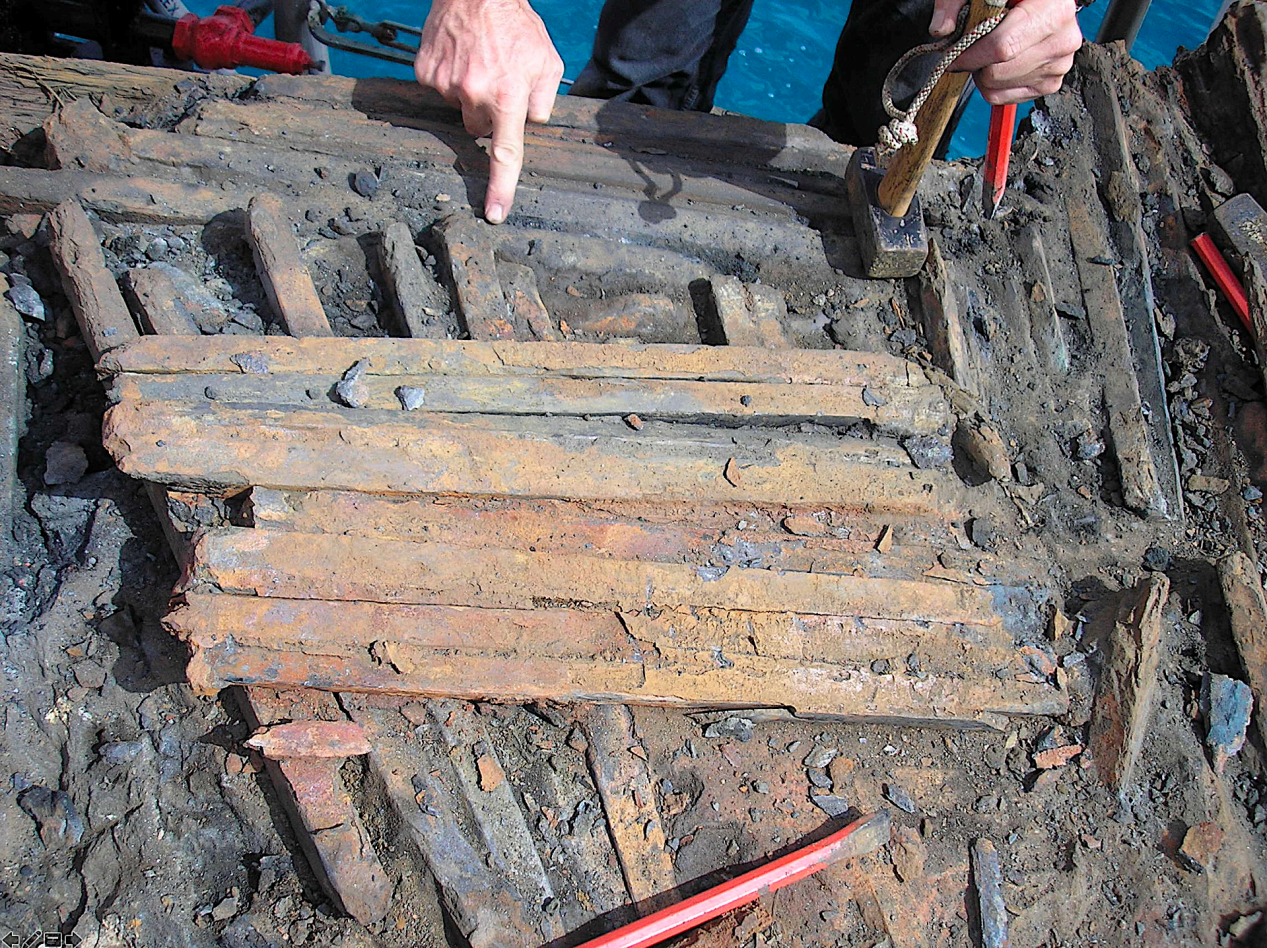
Small portion of a cargo of iron bars carried on the Saintes-Maries-de-la-Mer wrecks (pictured here, type-2M bars from the SM25 wreck).

The Rhône River was a major axis of communication linking maritime trade from the Mediterranean to the provinces of Gaul, Britannia and Germania. Its many branches and ports around its delta facilitated the development of impressive volumes of trade. At the outlet of the middle branch (Saint-Ferréol Rhône), 45 wrecks were discovered, opposite Saintes-Maries-de-la-Mer (Bouches-du-Rhône, France). The majority of the vessels (31 out of 45) carried cargoes of metal (Fig 1), and 23 transported only iron in the form of bars (Fig 2). To date, these constitute the largest assemblage of artefacts of Roman iron trade, attesting to the importance of this trade route, despite the risk of shipwreck in the delta in particular caused by offshore bars [3,4].

At the same time, Gaul was certainly the region in the Western Roman Empire with the largest iron deposits from the 2nd century BC to the 2nd century AD [5,6]. Huge mining operations in Gaul produced hundreds of tons of iron per year. From north to south, the iron production areas of Entre-Sambre-et-Meuse (Wallonia, Belgium), Sénonais-Pays-d’Othe (Yonne, France), Puisaye (Yonne and Loiret, France) and Montagne Noire (Aude, France) have been recognized in literature [5,6]. According to results of recent research programs that will partly be presented in the present paper (cf. Funding for the detailed list of these programs), Eastern Condroz (Wallonia, Belgium) in northern Gaul and Canigou (Pyrénées-Orientales, France) in southern Gaul can be added to the list for a total of six main iron-producing areas.

It follows logically that the Saintes-Maries-de-la-Mer wrecks and these six iron production areas in Gaul must be considered together in a study of the iron trade in the western part of the Empire. Such an investigation will also provide information about the geographical scope to be taken into account when sourcing antique iron, especially in Europe (such as whether a continental or regional approach is most relevant). It will also inform the sampling strategy to be implemented to meaningfully determine the chemical signature of a production area encompassing a very large number of smelting sites.

These last decades, provenance studies of iron artefacts were developed by several research teams [7,8].They are based on two complentary approaches. The first one considers that the chemical signature of Rare Earth trace Elements (i.e. their respective ratios) of the initial ore and more widely of the reduction system (i.e. ore, furnace lining etc) is preserved from the slag discovered on the smelting sites to the fragment of these slag that remain entrapped under the form of inclusions in the metallic matrix of the produced metal. Coustures *et al* [9] were the precusors of this approach but it was significantly refined particularly by introducing statistical data treatments of the results to examine the provenance hypothses [9–13]. The second approach is based on the principle that the isotopic signature of some elements initially present in the ore is preserved in the metal. Here, the isotopic signature of the metal of the artefact is compared to the one of potential ores. These last years, studies using the Os [12,14,15] and more recently Fe isotopes [16,17] were implemented with a relative success. Both approaches present their pro and cons. The “slag inclusion/trace elements” approach considers between 10 and 20 elements, allowing a powerfull discrimination between the potential sources. Unfortunately it needs to perform invasives sampling and also sometimes complex statistical approaches. The isotopic approach allows one to consider directly the metal and not the slag inclusions entrapped in it, but is far less disctriminant than the first approach because of the overlapping of the isotopic signature between different ores and region (for Fe isotope see for example [18]). Moreover, reference data are not so numerous up to now for the isotopic approach.

Considering that the most effective approach is the one based on trace elements in slag inclusions, especially because a high number of reference sets of slag were already analysed and are available for comparison, this paper aims to set a new milestone in the traceability of iron in the western Roman Empire, breaking new ground by performing a first-ever statistical analysis of the data on this same set of artefacts (the iron bars of the Saintes-Marie-de-la-Mer wrecks), based on 12 trace chemical elements, as well as analyzing four additional iron bars, for a total of 13 iron bars. Moreover, this article publish for the first time recent results from research programs on Gallo-Roman iron production areas (see Funding part for the detailed list of these programs), making it possible to analyze an extensive collection of more than 120 smelting slags from six production areas, which constitute a yet unpublished chemical database for determining the origin of iron in the western Roman Empire. In a first stage, the compositions of the smelting slag found in the considered production areas are compared. Then, these compositions are compared to the one of the slag inclusions entrapped in the artefacts, or different parts constituting an the artefact (called “Primary Pieces of Metal: PPM). Actually some bars are only formed by one single PPM, some other types of bars are made by welding togheter several PPMs (cf. *infra* and [19]). Thus PPMs constituting a given bar can potentially have different provenances. This comparisons will allow us to distinguish different provenance groups for the artefacts (caracterised by a similar trace element chemical composition). Some of these groups are compatible with the composition of the slag of some of considered production areas (i.e. areas where the iron ore is smelted into metal). Lasltly, these archaeometric results are discussed by confrontation with archaeological considerations to propose a new vision of the exchange networks during the antiquity between Gaul and the Mediterranean.

## 2. Set of samples

### 2.1. Production areas

**Fig 3:**
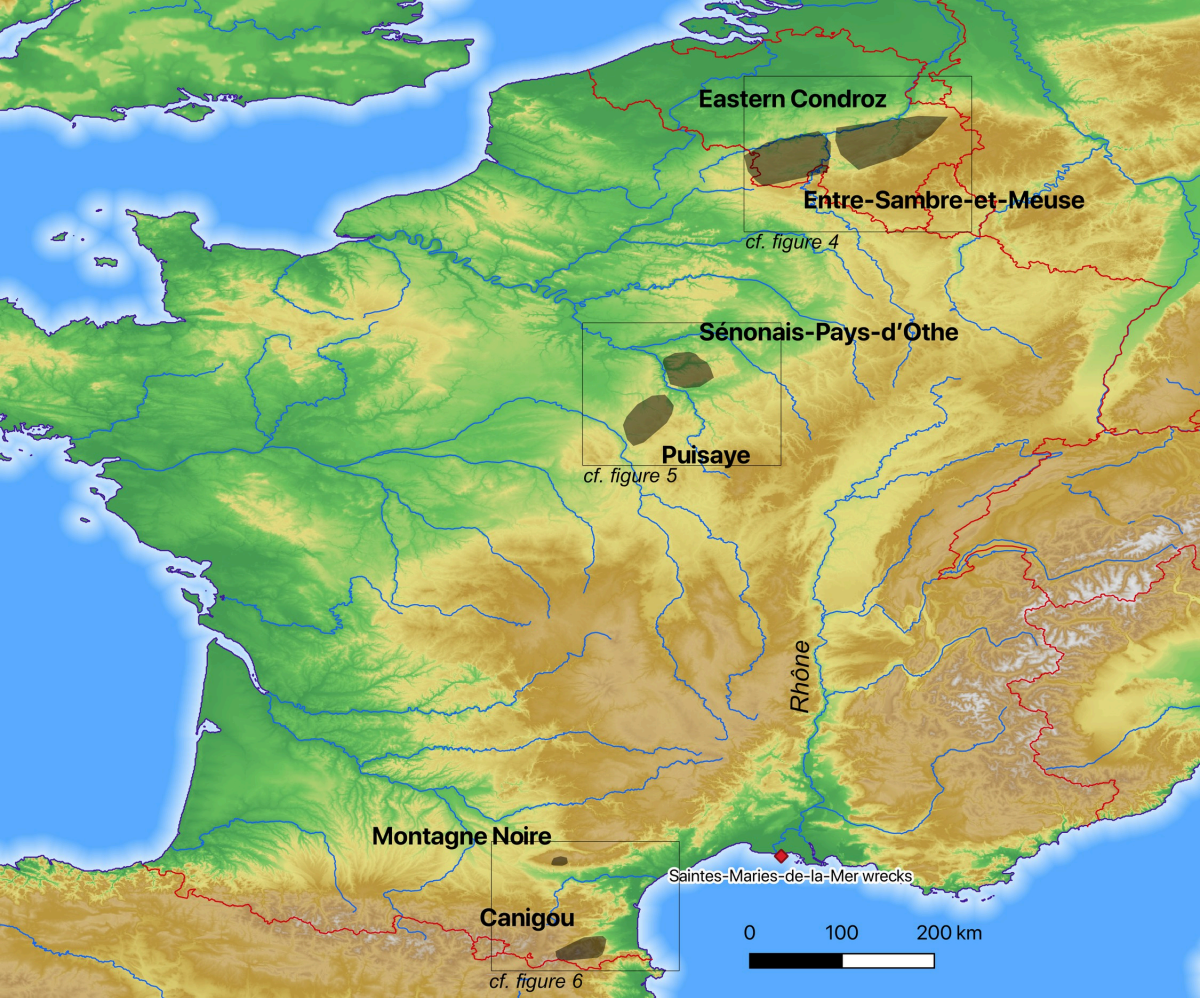
Locations of the iron production areas (north at the top; DEM NASA Shuttle Radar Topography Mission Global 1 arc second).

Tapped slag was collected from the six territories in western Europe considered to be among the largest iron-producing areas in the Roman period (Table 1, Fig 3). From north to south, they are currently located in Wallonia, in Belgium (Eastern Condroz and Entre-Sambre-et-Meuse), in central and southern France (La Puisaye, Sénonais-Pays-d’Othe, Montagne Noire and Canigou).

**Table 1:** Slag samples from the production areas.

Area	Site name	Abbreviation	Municipality	Region	Country	Datation	No. of samples
Eastern Condroz	Ferrières- Izier	Izier	Durbuy	Wallonia	Belgium	Antiquity/ early Middle Age	5
Eastern Condroz	Haye des Chènes No. 6 (or Autoroute)	Auto	Sprimont	Wallonia	Belgium	La Tène/ Roman Antiquity	7
Eastern Condroz	Haye des Chènes No. 7 (or Blindef)	Blindef	Sprimont	Wallonia	Belgium	La Tène/ Roman Antiquity	7
Entre-Sambre-et-Meuse	Bonnes Revaux—Hanzinelle	B_Revaux	Florennes	Wallonia	Belgium	La Tène/ Roman Antiquity	4
Entre-Sambre-et-Meuse	Pumont	Pumont	Walcourt	Wallonia	Belgium	Roman Antiquity	3
Entre-Sambre-et-Meuse	Froidchapelle-Géronsart	Geron_Froid	Couvin	Wallonia	Belgium	Roman Antiquity	3
Entre-Sambre-et-Meuse	Ferrière -Virelles	B_Ferriere	Chimay	Wallonia	Belgium	Roman Antiquity	3
Entre-Sambre-et-Meuse	Tienne Jacquet-Géronsart	Geron_Jacq	Couvin	Wallonia	Belgium	Roman Antiquity	3
Entre-Sambre-et-Meuse	Baterage	Baterage	Couvin	Wallonia	Belgium	Roman Antiquity	4
Montagne Noire	Laprade-Basse	MN-LB	Cuxac-Cabardès	Aude	France	Roman Antiquity	4
Montagne Noire	Co d’Espérou	MN-Esp	Saint-Denis	Aude	France	Roman Antiquity	4
Montagne Noire	Camp Naout	MN-Naou	Saint-Denis	Aude	France	Roman Antiquity	4
Montagne Noire	Carreleit	MN-Ca	Fontiers-Cabardès	Aude	France	Roman Antiquity	4
Montagne Noire	Domaine des Forges		Les Martys	Aude	France	70 BC– 270 AD	4
Montagne Noire	Monrouch	MN-MO	Les Martys	Aude	France	70 BC– 270 AD	4
Canigou	Coll del Forn	FORN	Estoher	Pyrénées-Orientales	France	Roman Antiquity	5
Canigou	Les Colomines	COLO	Taurinya	Pyrénées-Orientales	France	100 BC– 50 AD	4
Canigou	Camp del Pull 1	PULL-N	Saint-Marsal	Pyrénées-Orientales	France	125–25 BC	4
Canigou	Arles-sur-Tech village	Arles	Arles-sur-Tech	Pyrénées-Orientales	France	50–225 AD	3
Canigou	Oratori StMarsal	OR-M	Saint-Marsal	Pyrénées-Orientales	France	125–25 BC	4
Canigou	Pla de l’Abella 1	ABEL-F	Saint-Marsal	Pyrénées-Orientales	France	125–25 BC	4
Puisaye	Bois des Ferriers 1	F1-S3-301	Aillant-sur-Tholon	Yonne	France	42 BC– 235 AD	8
Puisaye	Bois des Ferriers 3	F3-S1-103	Aillant-sur-Tholon	Yonne	France	84–340 AD	4
Puisaye	Bois des Ferriers 9	F9-S1-101	Aillant-sur-Tholon	Yonne	France	Antiquity/ early Middle Age	2
Puisaye	Le Ferrier Guillou 1	Guillou 1	Dracy	Yonne	France	Antiquity	1
Puisaye	Le Ferrier Guillou 2	Guillou 2	Dracy	Yonne	France	127–325 AD	1
Puisaye	Jubin	Jubin	Lavau	Yonne	France	Antiquity	1
Puisaye	Les Gâtines Beauchet	89.420.001	Treigny 1	Yonne	France	717–393 BC	2
Puisaye	Bois des Chataigniers	89.420.002	Treigny 2	Yonne	France	401 BC -115 AD	2
Puisaye	Bois des Chataigniers	89.420.003	Treigny 3	Yonne	France	540–387 BC	2
Sénonais-Pays-d’Othe	Noslon	Noslon	Cuy	Yonne	France	La Tène C2/D1	9
Sénonais-Pays-d’Othe	Defendable	Defendable	Villiers-sur-Seine	Seine-et-Marne	France	La Tène D	3
Sénonais-Pays-d’Othe	Les Fouetteries (Les Clérimois)	Les Fouetteries	Les Clérimois	Yonne	France	40 BC—592 AD	8

**Fig 4:**
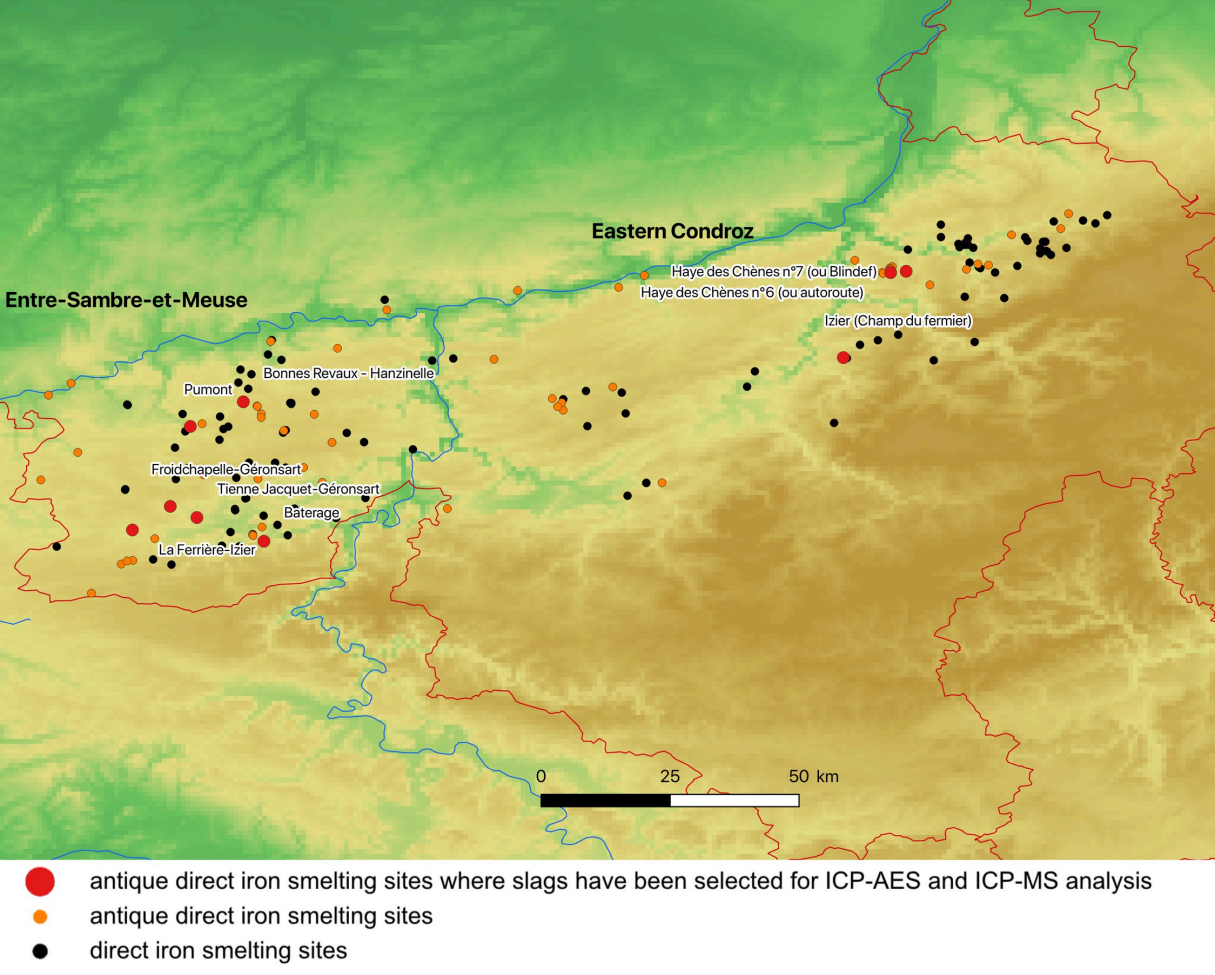
The Entre-Sambre-et-Meuse and Eastern Condroz iron production areas in Wallonia (Belgium, north at the top; DEM NASA Shuttle Radar Topography Mission Global 1 arc second).

In Wallonia (Belgium), the Condroz is located between the Meuse river and the Ardennes massif, stretching 130 km from east to west (Fig 4). This geological entity contained large quantities of iron ore mined in ancient times, in the form of limonite, from clay-sand karst filling as well as from seams of oxidized sulfide ore, all of Famennian or Dinantian age [20,21]. The sedimentary deposits of the minette and oolitic oligist types present in the Paleozoic and Mesozoic rocks were exploited more recently mainly from the 19th century. In this area, based on bibliographic and field research, as many as 172 smelting sites (slag heaps) have been inventoried, by Vincent Serneels in the 1970s, Geoffrey Houbrechts [22] and Gaspard Pagès (during his post-doctoral work at Liege University between 2009 and 2011). They all contain tapped smelting slag typical of the bloomery (or direct) process. Among this 172 sites, sixty-four slag heaps were dated by ceramics from La Tène D to late antiquity [23–33]. Based on the number of sites, the volume of tapped slag and the estimated quantity of slag recycled in blast furnaces since the 19th century, more than one million tons of slag may have been produced during this period [34–36]. In the western Condroz, the Entre-Sambre-et-Meuse area can be distinguished by the number and the concentration of iron smelting sites and iron ore deposits (92 smelting sites, including 36 from the Roman period). The Entre-Sambre-et-Meuse area extends south into a geographical region called Fagnes, which is devoid of ore deposits, but features smelting sites. In this study, we therefore distinguish between Eastern Condroz (2,800 sq. km) and Entre-Sambre-et-Meuse (2,600 sq. km). 19 slag samples from three Eastern Condroz sites dated from the end of Antiquity and 20 slag samples from six Roman Entre-Sambre-et-Meuse sites were selected based on chronology, conservation and size.

**Fig 5:**
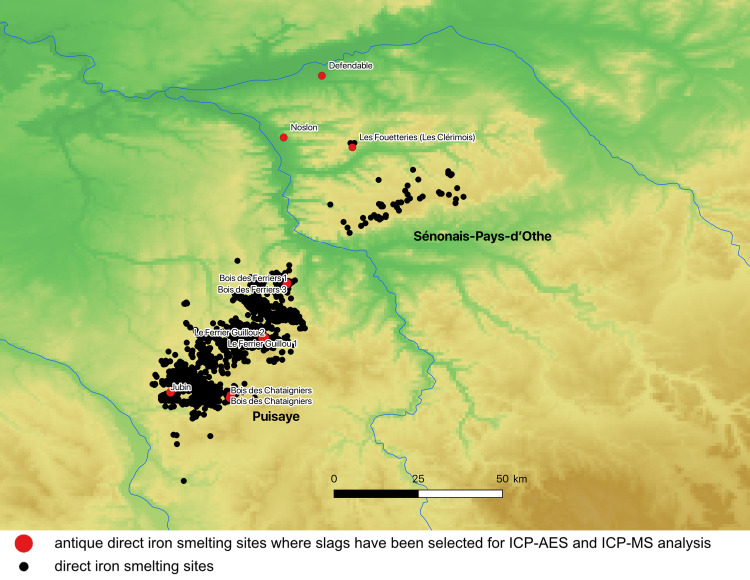
The Puisaye and Sénonais-Pays-d’Othe iron production areas in France (north at the top; DEM NASA Shuttle Radar Topography Mission Global 1 arc second).

Puisaye and Sénonais-Pays-d’Othe (Yonne and Seine-et-Marne, France) are contiguous regions along the southeast edge of the Paris basin and feature the same geology: low plateaus shaped in the clay and marl soils of the Tertiary Eocene age. They are rich in limonite deposits, particularly in the Sparnacian stages, and form iron concentrations of varying thickness, which were most likely significantly modified over geologic time. La Puisaye is the best-known region, due to a survey that was conducted systematically for over a decade starting in the 2000s [37], enabling the identification of more than 2,500 slag heaps in an area of 1,800 square kilometers (Fig 5). In 2017, a research program was launched to establish a detailed chronology of the evolution of smelting techniques. These sites are estimated to have produced several million tons of slag. Production developed here from protohistory to the Middle Ages, with a floruit during antiquity. Currently, Puisaye is the largest known iron production area in Gaul, especially during Roman times. From this area, 23 slag samples were analyzed, taken from three protohistoric sites and six Roman sites. Sénonais-Pays-d’Othe resembles Puisaye geologically and geographically, but is lesser-known [38,39]. This 1,500-sq. km region is probably an extension of Puisaye. It constitutes one of the largest metal-producing territories in Gaul, especially during Roman times. From this area, 20 slag samples from two La Tène sites and one Roman site were analyzed.

**Fig 6:**
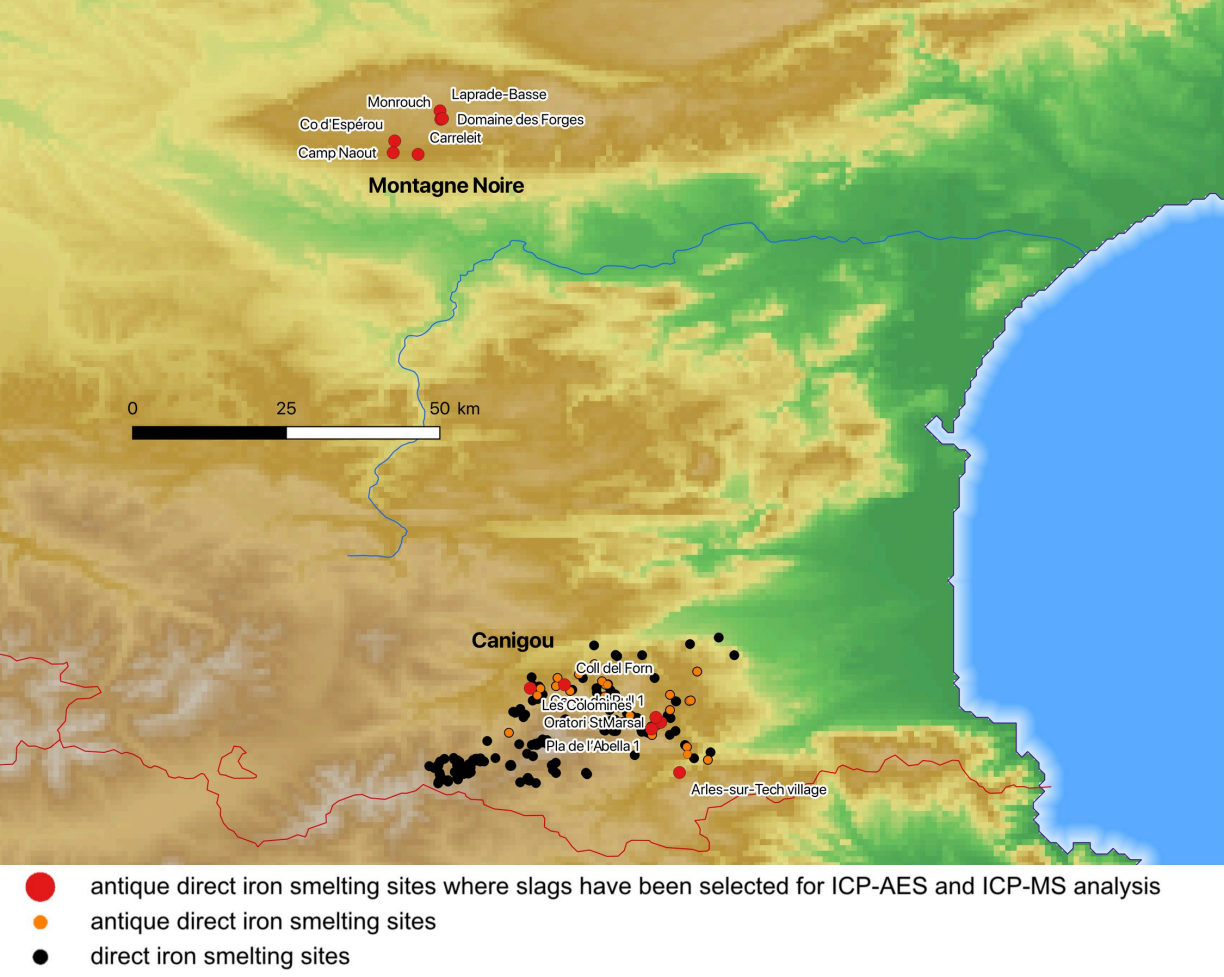
The Montagne Noire and Canigou iron production areas (north at the top; DEM NASA Shuttle Radar Topography Mission Global 1 arc second).

The southern side of Montagne Noire (Aude, France) is a granite-gneiss massif where ores derived from sulfide oxidation are localized in vein and stratiform structures of Cambro-Ordovician age [9,16]. They are mainly manganiferous and were processed at Roman smelting sites clustered above the Dure, Alzeau and Orbeil valleys [40]. In this area, 227 smelting sites were inventoried, including 28 Roman sites. Based on the volumes of the slag heaps, some 0.3 million tons of slag were produced in this area (Fig 6). Although this iron-producing area is not extensive (covering just 150 sq. km), it encompasses smelting sites of significant size, the best-known being Le Domaine des Forges (Martys, Aude, France) [9]. Trace element analyses for Montagne Noire have been published in previous studies; however, these mainly considered iron ore and not slag. To date, only four analyses of production slag from this area have been published [9]. We decided to complete this set of data with new analyses of tapped slag samples from an additional five Roman sites, for a total of 24 analyzed slag samples from this production area.

The Canigou Massif (Pyrénées-Orientales, France) is a mountain with a complex geology that culminates at 2,980 meters above the Mediterranean Sea (Fig 6). Large deposits of manganiferous iron ores are present on its slopes. These are geologically similar to the Montagne Noire deposits. They were formed by hydrothermal circulation of fluids, infiltrating Cambrian and Ordovician volcano-sedimentary rocks along faults. The ores are generally in the form of oxides (hematite) and carbonates (siderite). In this massif covering 1,000 square kilometers, 262 smelting sites were discovered in the course of a research program that is currently in progress, cf. Funding and [41]. More than 0.4 million tons of slag were produced by 33 Roman smelting sites. From six of these Roman sites, 24 samples of slag were analyzed.

### 2.2. Artefacts: Iron bars of Saintes-Maries-de-la-Mer (Bouches-du Rhône, France)

**Fig 7:**
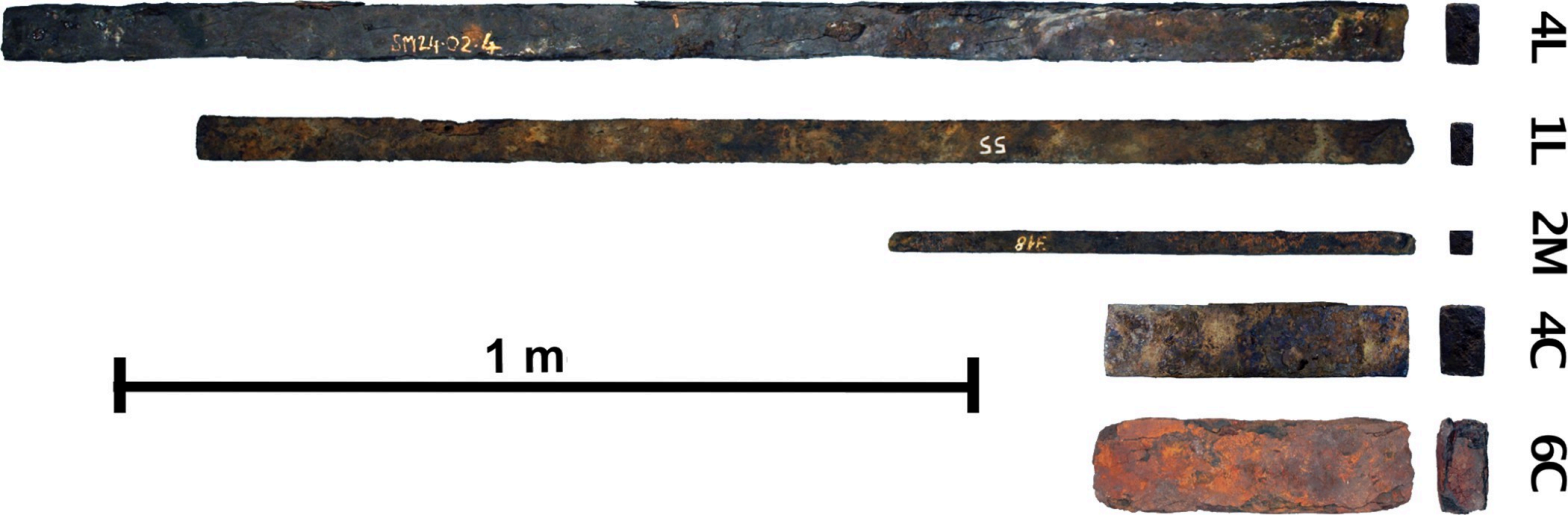
Bar types studied here. For further information on typology, see [3,19].

The list of analyzed bars is provided in Table 2. The analyses take into indicates the iron bar types defined by L. Long [3] (Fig 7), as well as the types of marks stamped on some of the bars. In addition to original analytical results, the present study also includes published results from previously analyzed bars [9,40] in the statistical analyses. A first set of these earlier studies considered a limited number of trace elements and only bivariate comparisons to test the provenance hypotheses (despite a higher number of element was analysed and published). They attribute some of these bars to Montagne Noire or other unknown provenance groups (Table 2).These results are reconsidered here, using a statistical data treatment. In addition, some of these former studies presented a too low number of analyzed elements (to be really discriminant) or data that are too dispersed for meaningful comparison. For this reason, the present study only takes into consideration published results on bars for which a sufficient number of elements were previously analyzed. These previously published artefacts have been renamed here, in an effort to clearly indicate their type and the number of the wreck on which they were found. The name of all bars is now composed of the “type code” (1M, 2M, etc), then comes the “ship name” (SM1, SM2, …) and a an identification number. Then comes a letter identifying te laboratory where the bar was analysed (T: bars analysed at the Toulouse (France) laboratory by Coustures; L: bars analysed in the LAPA laboratory). Finally a PPM number if more than one PPM constitutes the bar. Indeed, as said before, some of the bars are composed of several Primary Pieces of Metal (PPM) that are welded together in a given workshop to obtain the final bar. These PPM could potentially comes from different production places, not the same than the one of the assembling workshop (and therefore have a different trace element chemical signature in their slag inclusions) [19]. That is why we choose to consider PPMs as separate entities in the present study. Unfortunately, no information on PPM number per bar is provided in earlier published studies dealing with trace elements [9,40]. For this reason, we will consider that these artefacts are potentially composed of several PPM, and procced to an initial sorting of the raw data to see if different clusters of signature (i.e. PPM) can be distinguished on the same bar. In addition to this set of bars, we will study a second set for which metallographic observations and a major element analysis of the slag inclusions have already been published [19]. Again, for these bars, each previously identified PPM were considered individually. In Pagès 2011 [19], each PPM was assigned to a family according to the major composition of its slag inclusions (Al_2_O_3_/SiO_2_ and MgO/Al_2_O_3_ ratios, presence of P_2_O_5_ or MnO). Ultimately, 13 bars were selected for the present study, of which four contained more than one PPM. As a result, a total of 22 PPM were analyzed. They come from five wrecks of different dating: SM2 (0–25 AD), SM6 (240–51 BC), SM9 (196–69 BC), SM10 (1 BC -215AD), SM24 (120 BC—75 AD) [2,42] (Fig 1).

**Table 2:** List of analyzed artefacts.

Name	Type	Stamp	Formerly published name	Publication reference	Previously assigned group
1L SM2 T1*	1L	Type 9: *IVL//EROTIS*	SM2-2-Y1	[9,40]	Coustures-G2 (MN)
1L SM2 T2*	1L	Type 9: *IVL//EROTIS*	SM2-96-KL-Y2		Coustures-G2 (MN)
2M SM6 T1*	2M	None	SM6-1-E2		Coustures-G1
2M SM6 T2* (A et B)	2M	None	SM6-2-E		Coustures-G1
2M SM6 T3*	2M	None	SM6-3-E2		Coustures-G1
2M SM9 T1* (A et B)	2M	None	SM9-99-248		Coustures-G1
4C SM2 T1*	4C	Type 9: *IVL//EROTIS*	SM2-4-B		Coustures-G2 (MN)
4C SM2 T3*	4C	Type 10: *S//LEPIDI//N*	SM-2-3-A-1		Coustures-G3 (MN)
6C SM6 T1*	6C	None	SM6-4-B-1		Coustures-Gind_2
1L SM10-2-L1	1L	None	1L SM10-2	[19]	Pages-Family 6 ** No MnO no P_2_O_5_ in slag inclusions
1L SM10-2-L2	1L	None	1L SM10-2		Pages-Family 3** High MgO in slag inclusions
1L SM2-1-L2	1L	None	1L SM2-1		Pages-Family 6** No MnO no P2O5 in slag inclusions
1L SM2-1-L3	1L	None	1L SM2-1		Pages-Family 6** No MnO no P_2_O_5_ in slag inclusions
4L SM24-1-L1	4L	None	4L SM24-1		Pages-Family 2** High P_2_O_5_ in slag inclusions
4L SM24-1-L3	4L	None	4L SM24-1		Pages-Family 2** High P_2_O_5_ in slag inclusions
4L SM24-1-L4	4L	None	4L SM24-1		Pages-Family 2** High P_2_O_5_ in slag inclusions
4L SM24-2-L1	4L	None	4L SM24-2	[19]	Pages-Family 2** High P_2_O_5_ in slag inclusions, high P in the metal
4L SM24-2-L2	4L	None	4L SM24-2		Pages-Family 6** No Mn no P_2_O_5_ in the slag, slow amounts of P in the metal
4L SM24-2-L3	4L	None	4L SM24-2		Pages-Family 1a** High MnO No P_2_O_5_ in slag inclusions
4L SM24-2-L4	4L	None	4L SM24-2		Pages-Family 1a** High MnO No P_2_O_5_ in slag inclusions

* Analyzed by [9,40].

** Families assigned based on major element analysis in slag inclusions [19]. MN: Montagne Noire.

## 3. Analytical methods

Representative samples of at least 3 cubic centimeter were taken using a diamond saw on the smelting slag found on the production areas (Figs 4–6, Table 1). They were then ground to a powder, with a grain size of about 70–80μm. Major and trace elements were quantified using inductively coupled plasma atomic emission spectroscopy (ICP–AES) and inductively coupled plasma mass spectrometry (ICP–MS), respectively, at CRPG Nancy France [43].

Concerning metallic bars, cross-sections were made on the complete artefacts using a SiC cutting disc. After a first grinding and polishing (SiC papers grade 180 to 4000), the welding lines indicating that a given bar is constituted of several Primary Pieces of Metal (PPM) welded together are searched. If they are detected, sub-samples are taken on each PPM. Otherwise, only one sample is taken. In a second step, samples are polished (diamond paste 3 and 1 μm under ethanol). This allows an observation by Optical Microscope and Scanning Electron Microscope to locate and analyse the non-metallic slag inclusions (made of a mix of silicates, oxides and vitrous phases) entrapped in the metallic matrix. The major element composition is determined by Energy Dispersive Spectrometry coupled to SEM (a FESEM JEOL 7100 equipped with an Oxford silicon drift detector, at 15kV with a probe current of 20nA). Prior to any spectrum acquisition, SI were detected by means of image analysis of the backscattered electrons signal. Particle detection and spectra processing were performed using the Aztec Software (Oxford Company). On each spectra the background was substracted using a top hat filter. The XPP correction routine was used for quantification (Phi-Rho-Zed matrix correction with inner standards). A fixed set of elements composing the iron slag is considered (O, Na, Mg, Al, Si, P, S, Cl, K, Ca, Ti, V, Cr, Fe, Mn). Concentrations were normalized at 100%. The analytical methodology has been described in detail elsewhere [10–12,44]. An important step in this procedure is the identification of those inclusions deriving from the ore reduction stage (the only ones that can be compared with the slag found on the production sites). Actually during the manufacturing of the bar, especially when welding together different pieces of metal (PPM), or during the shaping of the bars, some fluxes (clay, sand,…) could be added and generate new families of inclusions [45]. To differentiate these different kinds of slag inclusions (SI), the procedure proposed by Disser [46–48] for this identification was followed. Families of inclusions are determined based on major elements, especially non-reduced compounds (NRC: MgO, SiO_2_, Al_2_O_3_, K_2_O, CaO). In the thermodynamic conditions that take place in shaft furnces, these oxides are not reduced and their respective ratio do not change [45]. Several tens of inclusions were analyzed. This allows by a statistical and spatial consideration to identify different inclusions families by their composition [48] and to select the ones not located in the welding zones. Some 10 inclusions deriving from the ore reduction stage were then selected for LA-ICP-MS analysis. The VG Plasma Quad PWSX setup coupled with an Nd: YAG laser (wavelength of 266 nm) at the Centre Ernest Babelon (IRAMAT UMR7065) was used for this purpose. Ablations lasted 50 seconds with laser frequency set at 7 Hz and ablation diameter set at 80 μm. The quantification method developed by Gratuze [49,50] and adapted for SI studies [10,51] was employed. Thirty-eight trace elements were quantified, with an accuracy error usually below 12%. The net recorded signal for each trace element was normalized using the NIST610, NIST 612 international standards [44].

The trace elements considered in this paper are Ce, Eu, Hf, La, Nb, Nd, Pr, Sm, U, Y, Yb and Cs. This list of elements is the result of a compromise between the desire to study a maximum number of rare earth elements and the fact that only a limited number of trace elements have been analyzed in published studies on iron bars that are also included in our comparison set. Following the procedure proposed by [10] and [12], trace element contents were normalized using a log ratio approach given by the equation:

$$X_{E}=ln(\left[E\right])-\frac{1}{N}\sum_{k=1}^{N}ln(\left[E_{k}\right])$$

Given [E_k_], only the elements that were quantified for the whole reference set were considered: Y, La, Ce, Sm and Eu. The resulting variables were named “xij”.

Statistical data analysis was performed on xij. The chosen multivariate analysis method was principal component analysis (PCA), sometimes in combination with hierarchical agglomerative clustering (HAC) (FactoMineR and factoextra packages in R software). For PCA, it is verified if the composition of the slag inclusions of a metallic artefact graphically match with the one of other artefacts or the one of the slag of the production sites on all the 2D projections on the PCA dimensions gathering a significant part of the cumulated variance (> 75% generally). For the HAC the chemical compatibility is confirmed when the maximum distance separating two slag from a production area is greater than that separating the inclusion of an object from the nearest slag. The advantages and limitations of this approach compared to alternatives have been widely discussed elsewhere [8,10,12]. This approach was preferred here for its relative “neutrality” as an unsupervised method, compared with other methods such as Linear Discriminant Analysis, which may result in the incorrect grouping of artefacts into separate classes (for example, artefacts from the same reduction system–the same ore, furnace lining and charcoal–may be incorrectly separated because they come from two different smelts). These are aspects that should be investigated in more depth in the future.

## 4. Sorting of raw data on slag inclusions

**Fig 8:**
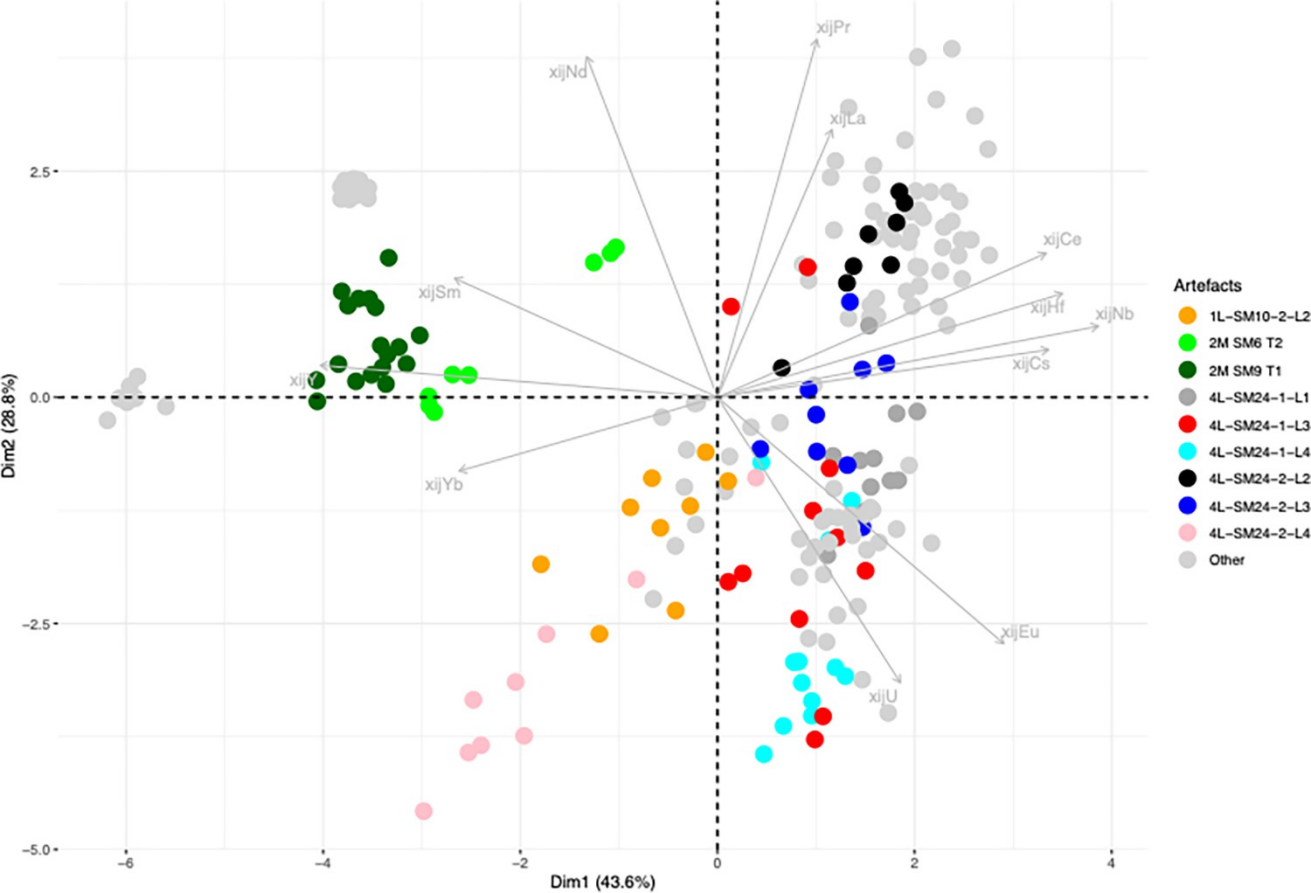
PCA analysis on xij (scaled) from the SI of the bars. Projection on the components 1 and 2 of the PCA.

The analytical results for the slag and the slag inclusions (SI) can be found in the supplementary materials (S1 Table). For this analysis, the raw data on slag inclusions were compared using PCA on scaled xij (Fig 8). When plotted, the inclusions found in each artefact form relatively well-grouped clusters. Nevertheless, there are some exceptions (shown in color in Fig 8), which will be discussed here individually.

The xij of the slag inclusions in 2M SM6 T2 form two distinct clusters. We therefore chose to consider that these two clusters represent two different PPMs constituting the same bar. They will be analyzed separately and hereafter referred to as 2M SM6 T2A and 2M SM6 T2B. For 1L-SM10-2L2, three inclusions can be considered to be outliers (1inc3, 2inc3 and 3 inc3 in the supplementary materials). For bar 2M-SM9 T1, one inclusion (248–47) can be considered to be an outlier, while the other inclusions form two clusters and will be hereafter referred to separately as 2M-SM9-T1A and B. For 4L-SM24-1-L1, one inclusion was discarded (2inc1). Four inclusions are outliers for 4L-SM24-1L3 (1inc2, 1inc3, 2inc2, 2inc 3). Three inclusions are outliers (1inc3, 4inc3, 2inc1) for bar 4L-SM24-1-L4, one inclusion is an outlier (1inc2) for 4L-SM24-2L2, two inclusions are outliers (1inc2, 3inc2) for 4L-SM24-2-L3 and two are outliers (2inc3, 1inc2) for 4L-SM24-2-L4.

## 5. Results

### 5.1. Production areas

**Fig 9:**
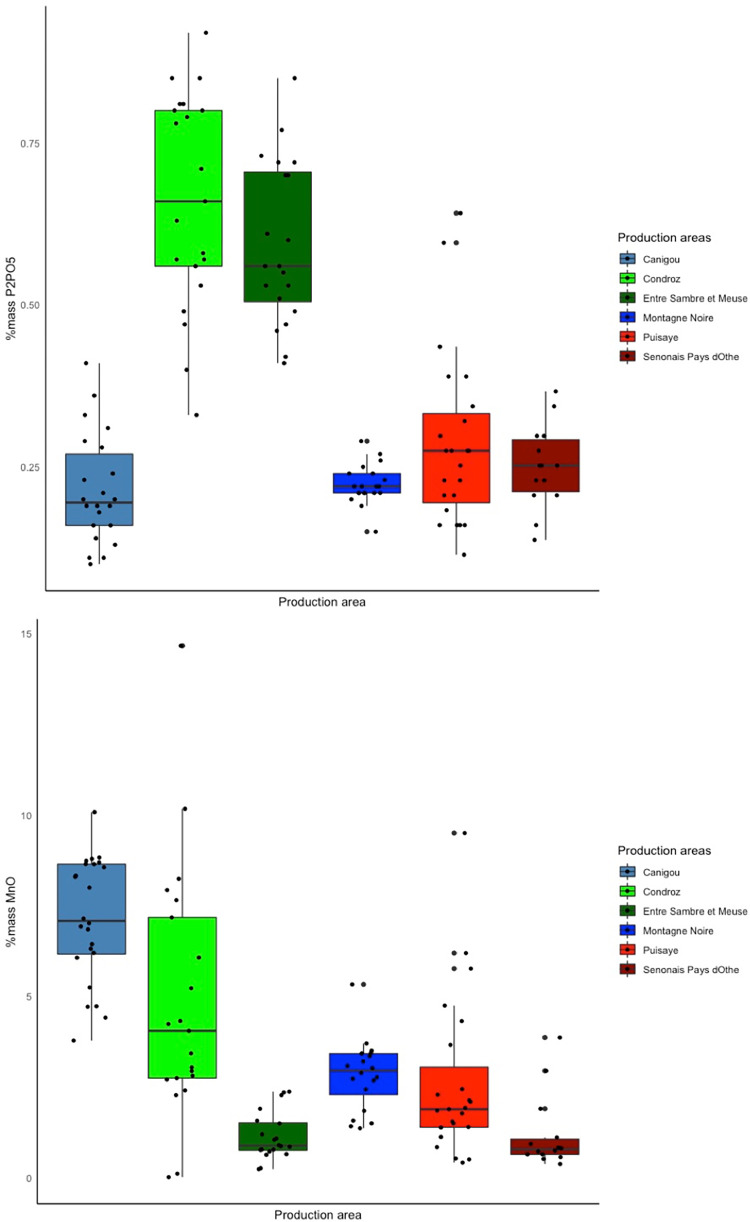
MnO and P_2_O_5_ content of slag from the production areas.

As far as major elements are concerned, P and especially Mn presence in the slag are mainly linked to the initial ore composition. Contrary to other elements (Si, Al, Ca,..) that are potentially linked to other sources (fuel ashes, lining of the furnace, etc). Thus P and Mn can be considered in a rought first step, to decipher different provenances [8]. Fig 9 shows the P_2_O_5_ and MnO contents of the slag samples from the different production areas. It is interesting to note that the MnO content of the slag is greater than 1% for four out of the six ironmaking areas: Canigou, Montagne Noire, Puisaye and Condroz. Therefore, in our case, the presence of this element in slag inclusions is not sufficient to suggest or determine a specific provenance. The P_2_O_5_ content of the slag from all the production areas studied here was below 1%, so it is very unlikely that metals or slag inclusions with high levels of phosphorus (e.g., 4L-SM24-1-L1, L3 and L4, 4L-SM24-2-L1) come from these areas.

**Fig 10:**
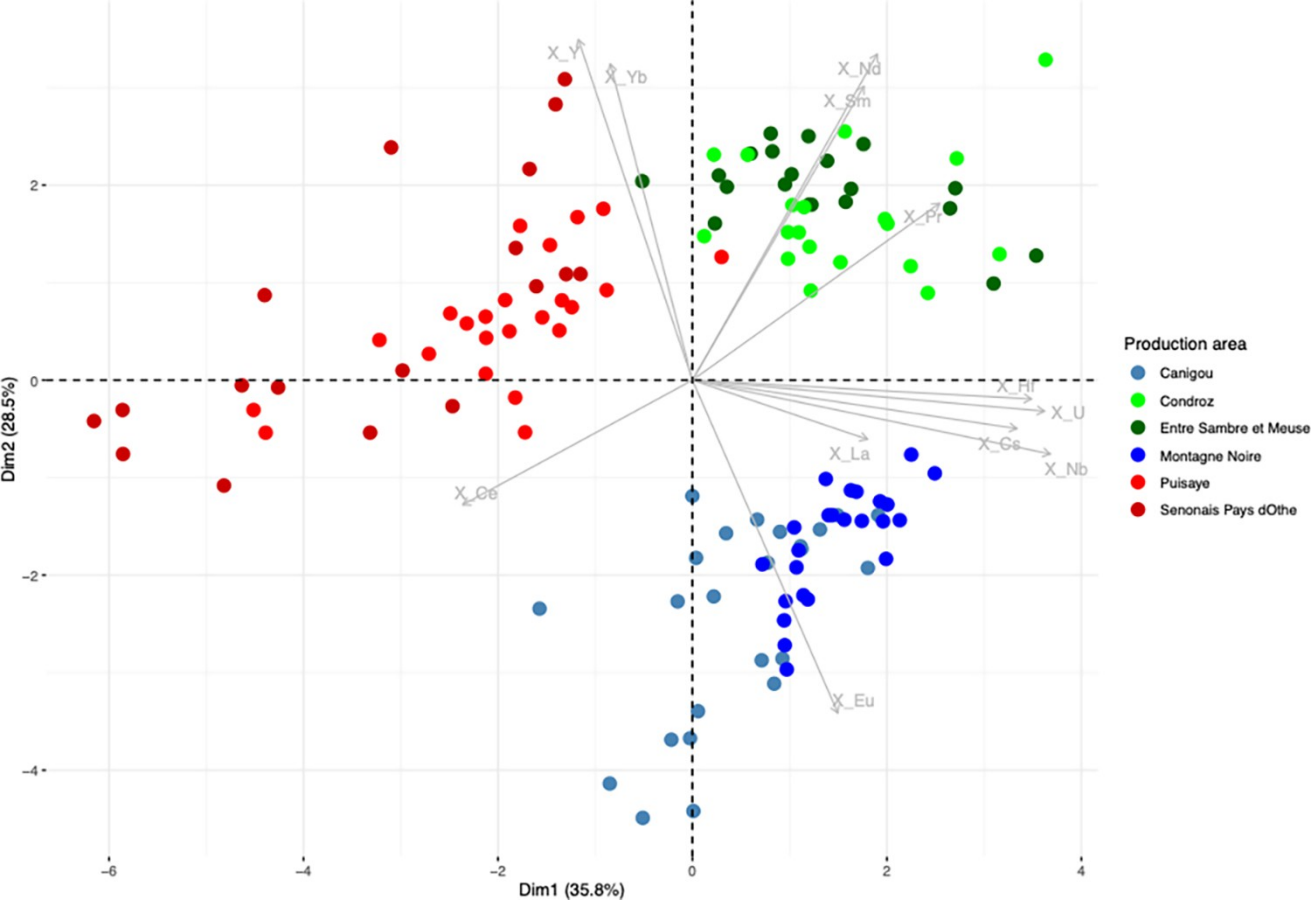
PCA on xij for slag from the production areas. % between brackets: % of total variance.

**Fig 11:**
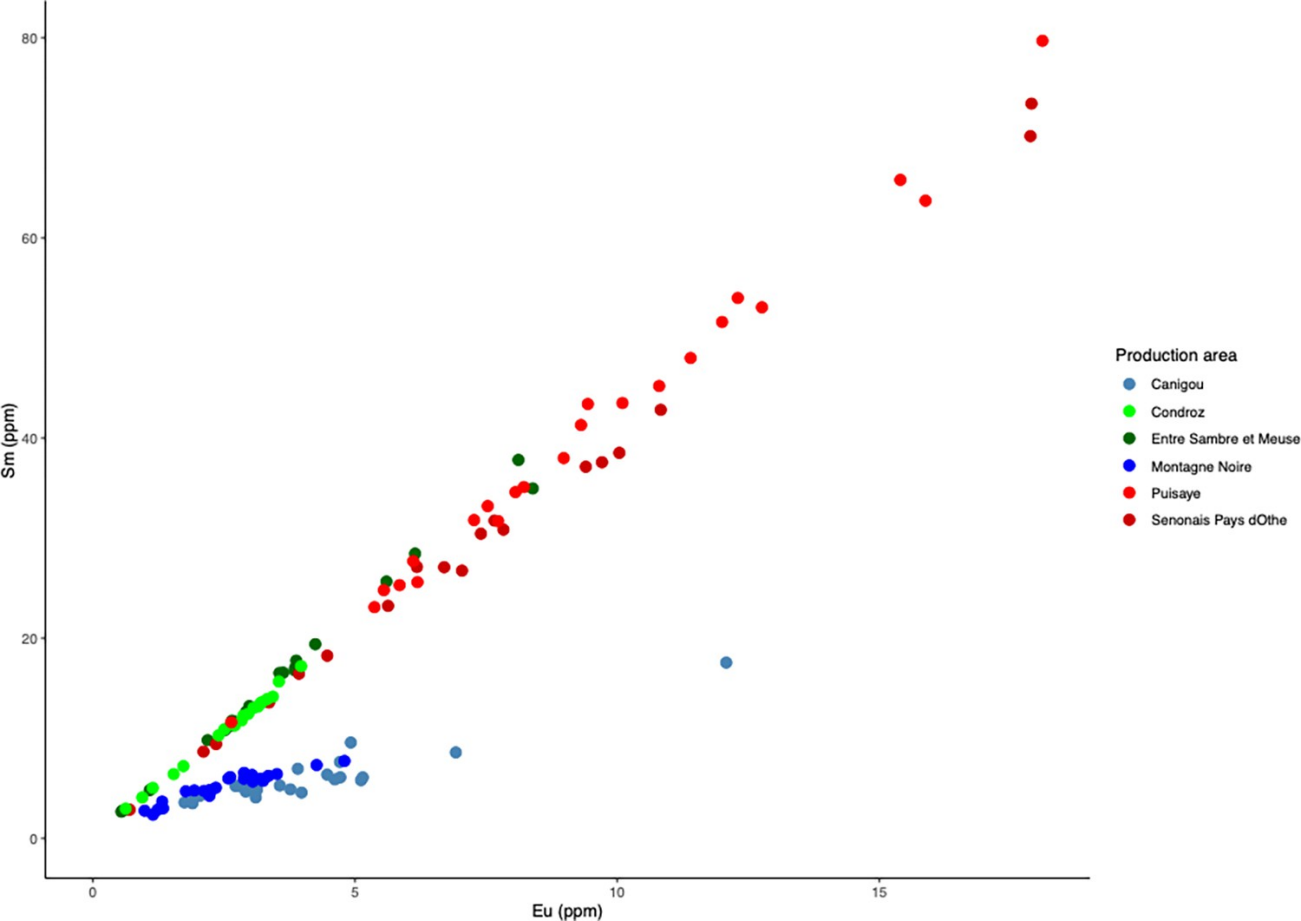
Eu and Sm content of the production area slag.

**Fig 12:**
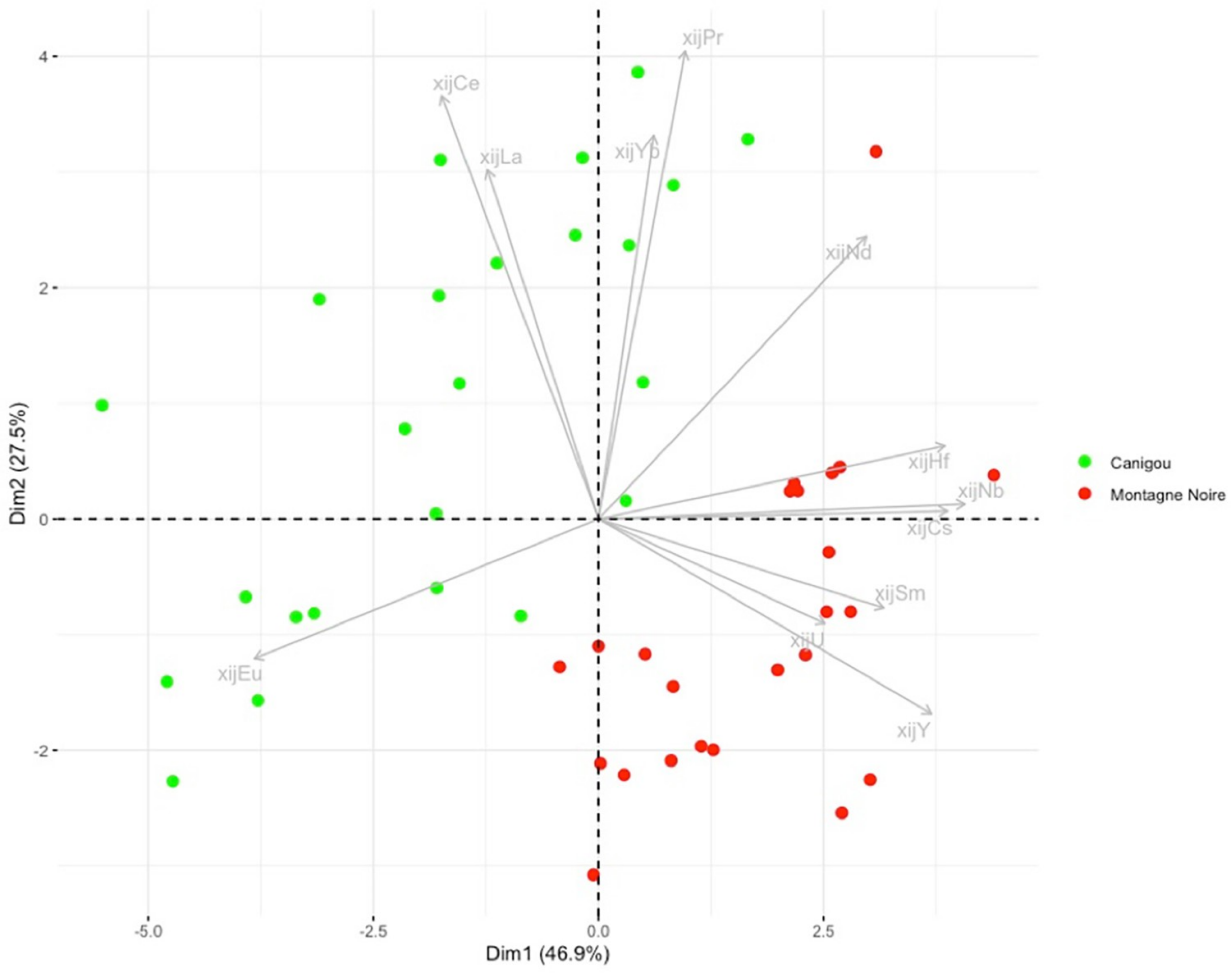
PCA on xij for slag from the Canigou and Montagne Noire production areas. % between brackets: % of total variance.

Let us now consider the trace element signatures of the production areas (Fig 10). In this Dim1/Dim2 projection, it can be seen that the six production areas are represented by three main clusters, roughly corresponding to the same number of geographical zones. The two production areas located in Wallonia (Belgium) plot in the same cluster, despite the difference of MnO content observed. Note that it is not possible to distinguish between these two areas using any PCA approach (including an analysis considering only the two production areas in Wallonia). A second cluster is formed by Sénonais-Pays-d’Othe and La Puisaye, two production areas that are relatively close to each other at the considered continental scale of the study. Again, these two production areas cannot be distinguished by PCA, even when only the two areas are considered. Lastly, Montagne Noire and Canigou are represented by the same cluster. It is worth to note that both areas show an anomalous Eu/Sm ratio (Fig 11), which was already observed for Montagne-Noire [52]. It is nevertheless possible to distinguish the two production areas when they are considered together using PCA (Fig 12).

### 5.2 Artefacts and production areas

**Fig 13:**
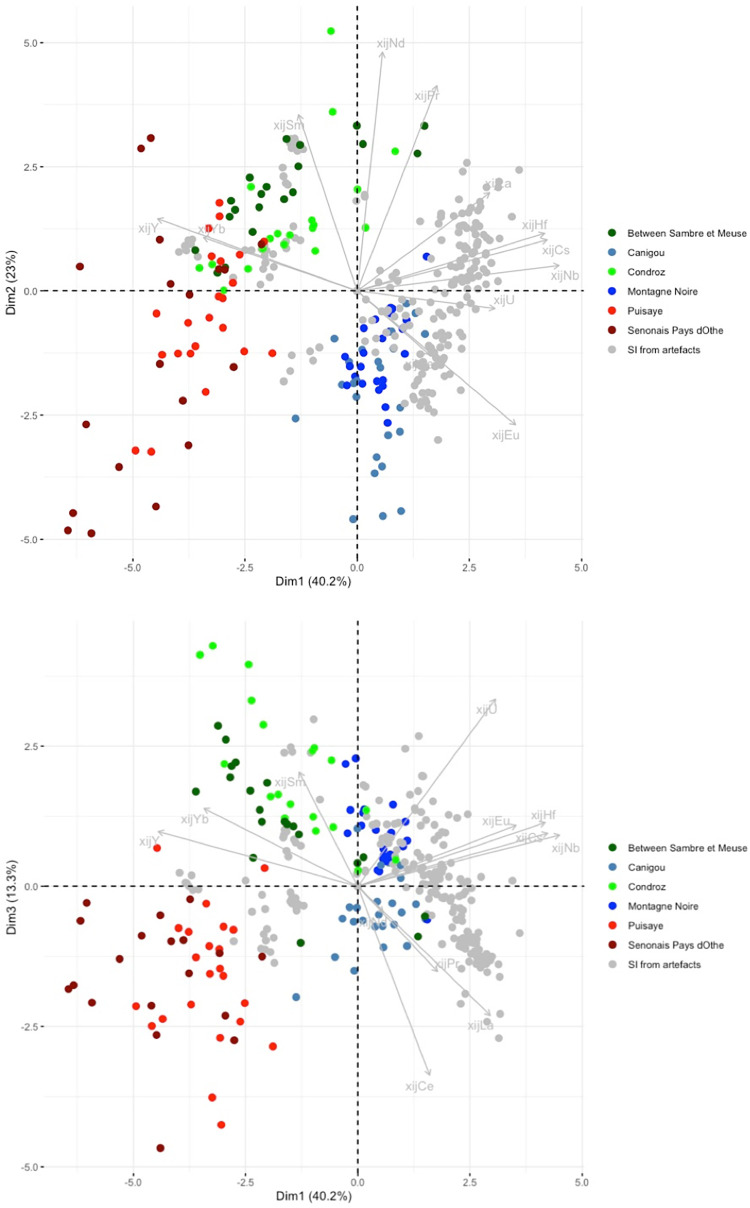
PCA on xij of production area slag. Only slag from production areas are highlighted. SI from artefacts are presented in grey. % between brackets: % of total variance.

For the first stage of statistical inference, we performed principal component analysis on the entire set of data (data from the slag samples from the different production areas and the slag inclusions embedded in bars and/or PPM). A first observation that can be made is that the three production area clusters are roughly preserved when slag inclusion data are added to the PCA (Fig 13).

**Fig 14:**
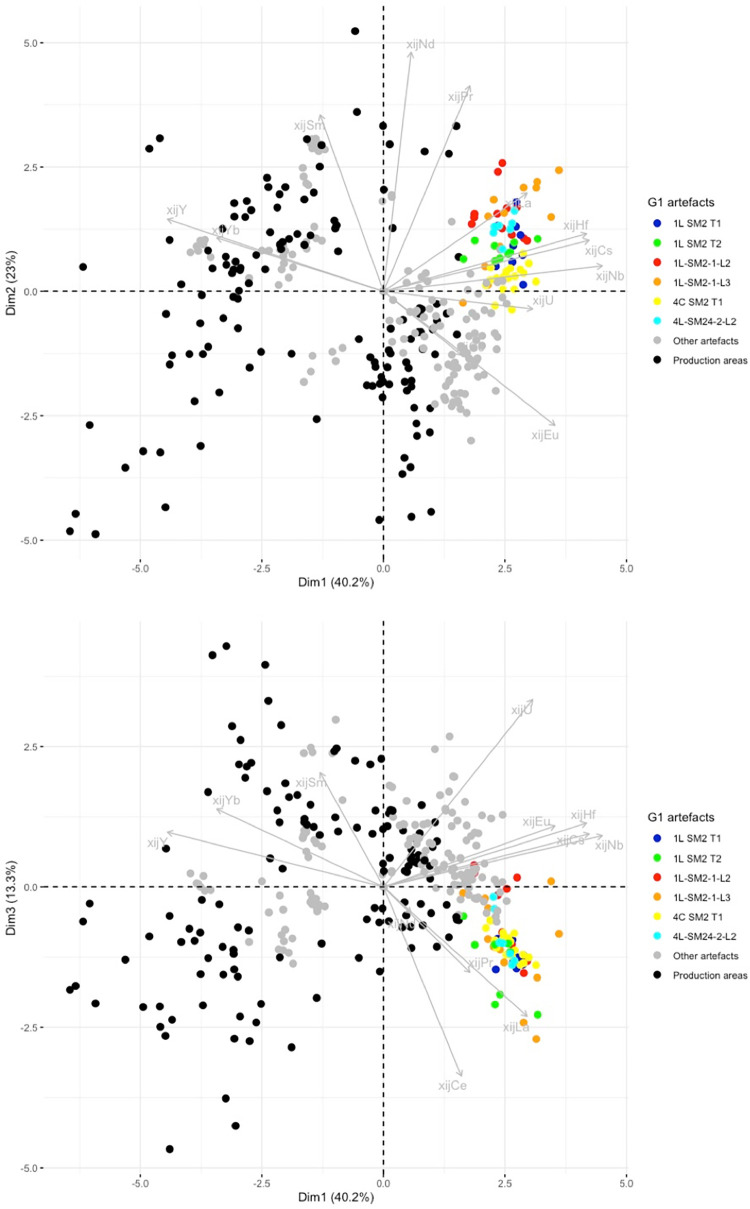
PCA on xij of production area slag. Artefacts of Group G1 are coloured (each colour corresponds to an artefact. Production areas are presented in black. % between brackets: % of total variance.

Some artefacts or PPM (defined by their inclusions) can be clearly graphically separated from the clusters corresponding to production areas, suggesting that they are not consistent with this provenance. A first group of artefacts (G1 in Table 3) is formed by the bars/PPM 1L-SM2-T1, 1L-SM2-T2, 1L-SM2-1L3, 4C-SM2-T1, 1L-SM2-1-L2, 4L-SM24-2-L2, which are all part of the same cluster in the projection of the first three dimensions of the PCA (Fig 14).

**Table 3:** Summary of the results of the statistical inference.

Name	Stage 1	Stage 2	Mark
1L SM2 T1	G1	G1	T9
1L SM2 T2	G1	G1	T9
2M SM6 T1	Sénonais-Pays-d’Hote/Puisaye	G5	None
2M SM6 T2A	Wallonia	Wallonia	None
2M SM6 T2B	Wallonia	Wallonia	None
2M SM6 T3	G4	G4	None
2M SM9 T1A	Wallonia	Wallonia	
2M SM9 T1B	G6	G6	
4C SM2 T1	G1	G1	T9
4C SM2 T3	Montagne Noire/Canigou	Montagne Noire	T10
6C SM6 T1	G2	G2	
1L SM10-2-L1	Montagne Noire/Canigou	Montagne Noire	
1L SM10-2-L2	Montagne Noire/Canigou	Montagne Noire	
1L SM2-1-L2	G1	G1	
1L SM2-1-L3	G1	G1	
4L SM24-1-L1	GP	GP	
4L SM24-1-L3	GP	GP	
4L SM24-1-L4	GP	GP	
4L SM24-2-L1	GP	GP	
4L SM24-2-L2	G1	G1a	
4L SM24-2-L3	GP2	GP2	
4L SM24-2-L4	G3	G3	

**Fig 15:**
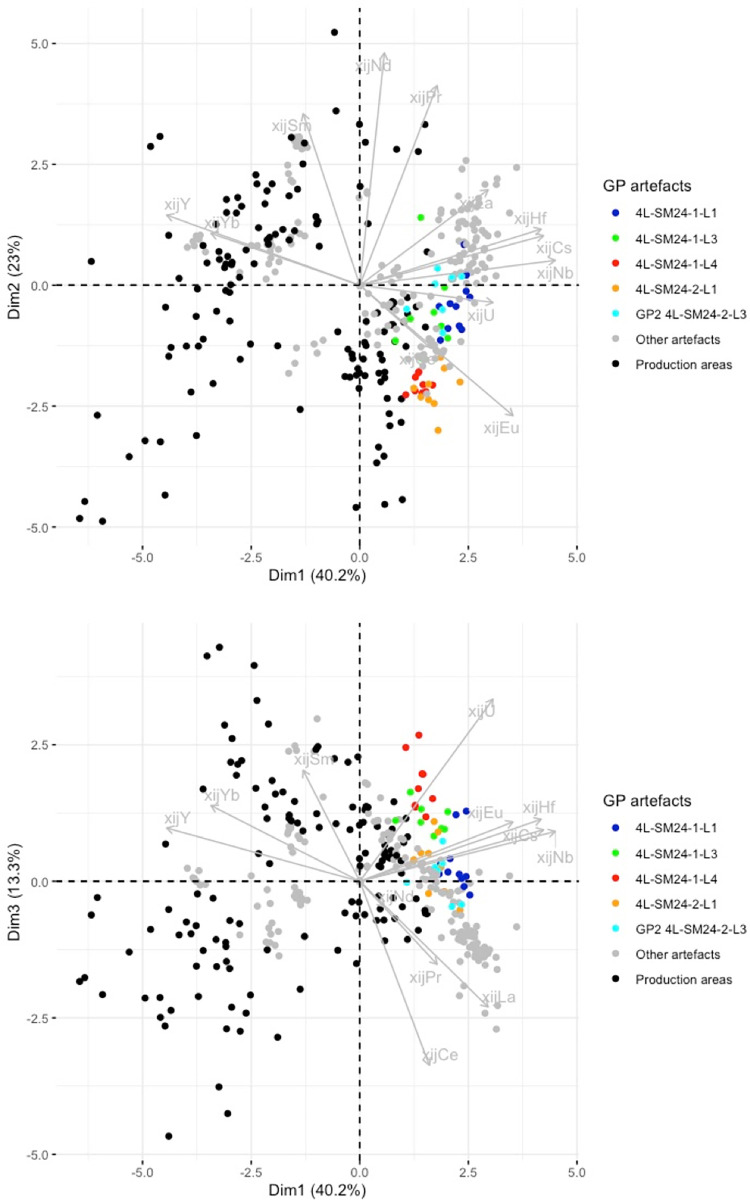
PCA on xij of production area slag. Artefacts of Group GP and GP2 are coloured (each colour corresponds to an artefact. Production areas are presented in black. % between brackets: % of total variance.

**Fig 16:**
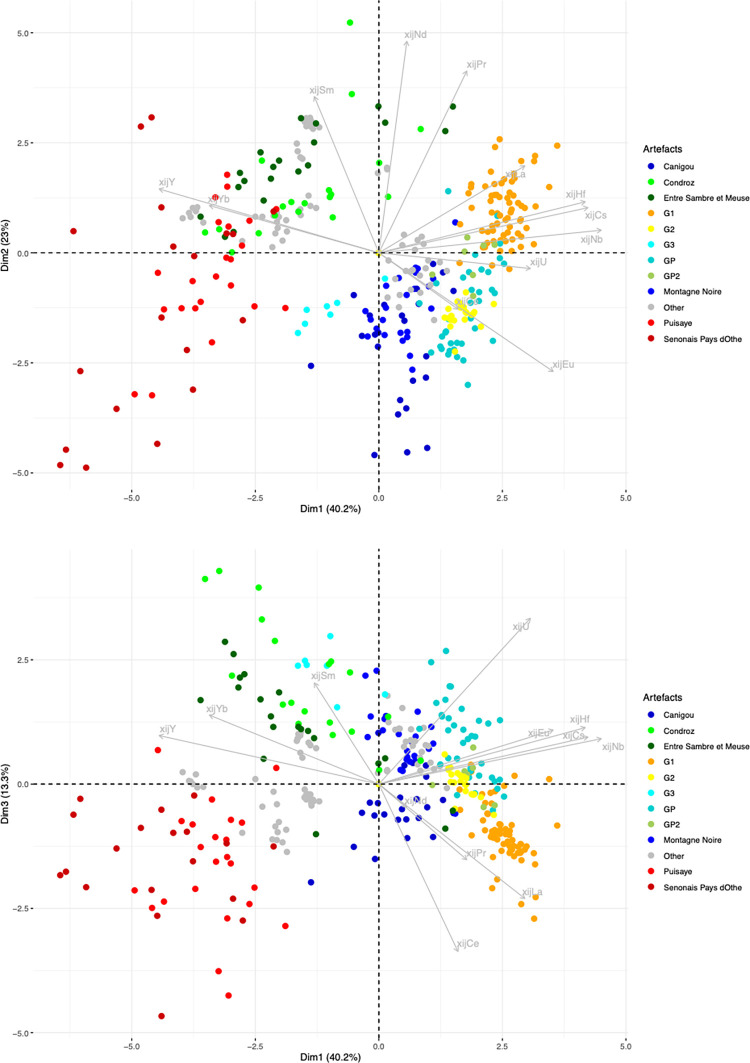
PCA on xij of production area slag. Artefacts not consistent with the production areas are highlighted. % between brackets: % of total variance.

In previous research [19], it was observed that 4L-SM24-1-L1, L3 and L4, and 4L-SM24-2-L1 are made of phosphoric iron and that their slag inclusions content very high amounts of phosphorus (between 8 and 10%). On the PCA projection, none of these bars fits with any tested production area but, despite a slight dispersion, their SI plot in the same area of the projections (GP on Fig 15). Consequently, considering the facts that the PPM all contain high levels of P in their slag inclusions and in the metal, and that they are located in the same area of the PCA projections, at this stage we consider them to be part of the same cluster (GP on Fig 15). PPM 4L SM24-2-L3 also plots in the same area of the PCA projection, but no P was detected in the slag inclusions by major element analysis. Therefore, at this stage we choose to identify it as a separate group (GP2). Another bar, 6C-SM6-T1, also plots in the GP and GP2 area of the projection. Unfortunately, this sample was not analyzed during the present study and there is no information in published literature on the presence of P in the slag inclusion. Hierarchical agglomerative clustering (HAC) performed on these six PPM (GP group, GP2 and 6C-SM6-T1) clearly shows that 6C-SM6-T1 is different from the five others (not shown). Therefore, we chose to place it in a separate group (G2). Lastly, 4L SM24-2-L4 (forming G3 group) does not match up with any production area or other artefact. Fig 16 shows the location of the different groups at the end of this first stage of the data analusis.

In the projection corresponding to the first three components (77% of the variance), the slag inclusions of some artefacts/PPM plot into the clusters corresponding to the production area (Fig 16). They will be considered in more detail below.

**Fig 17:**
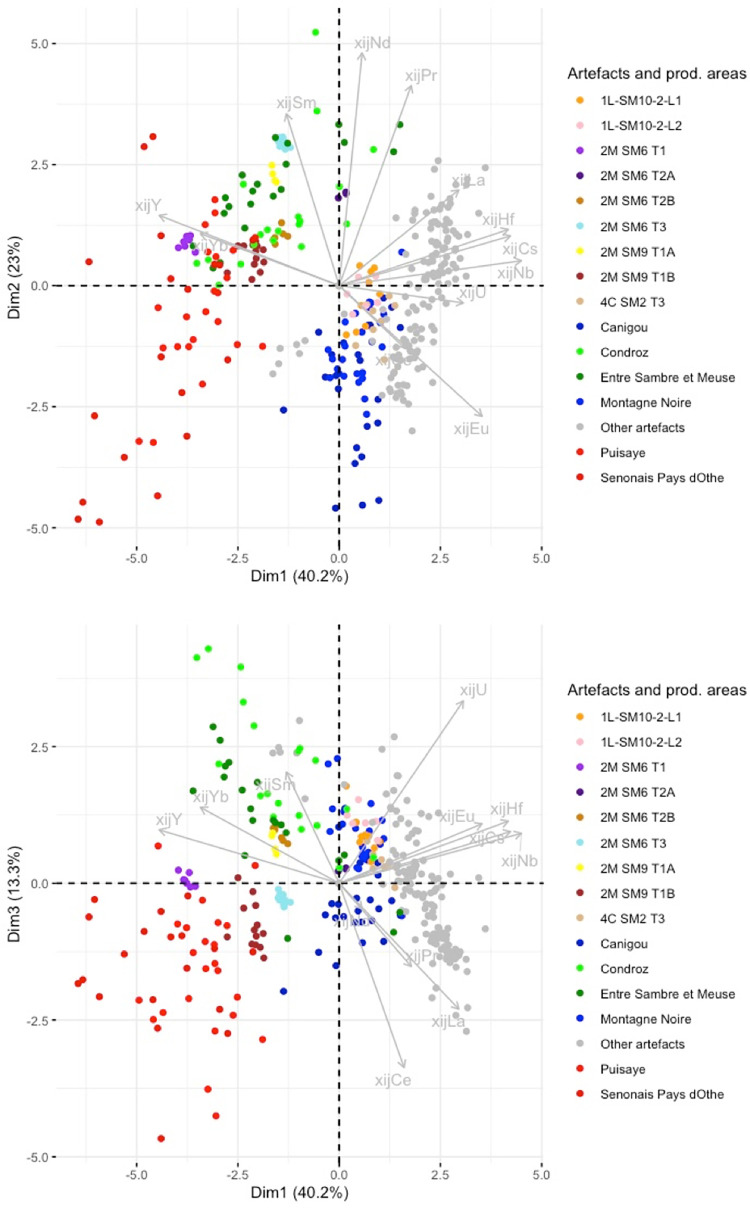
PCA on xij of production area slag. Artefacts consistent with a production area are highlighted. % of total variance.

Some artefacts are compatible with a particular production area on the Dim1/Dim2 projection but not on the Dim2/Dim3 projection. This is the case for 2M SM6 T3 and 2M SM9 T1B (Fig 17). They seem to correspond to two different unknown provenance groups (hereafter G4 and G6, respectively). Lastly, the slag inclusions of some artefacts plot in the same location in the Dim1/Dim2/Dim3 projection as the production areas. This is the case for 2M SM6 T1, compatible with Sénonais-Pays-d’Othe/Puisaye, 2M SM9 T1A, 2M SM6 T2A and T2B, compatible with Wallonia, and 4C SM2 T3, 1L SM10 2 L1 and L2, compatible with Montagne Noire/Canigou (Fig 17).

**Fig 18:**
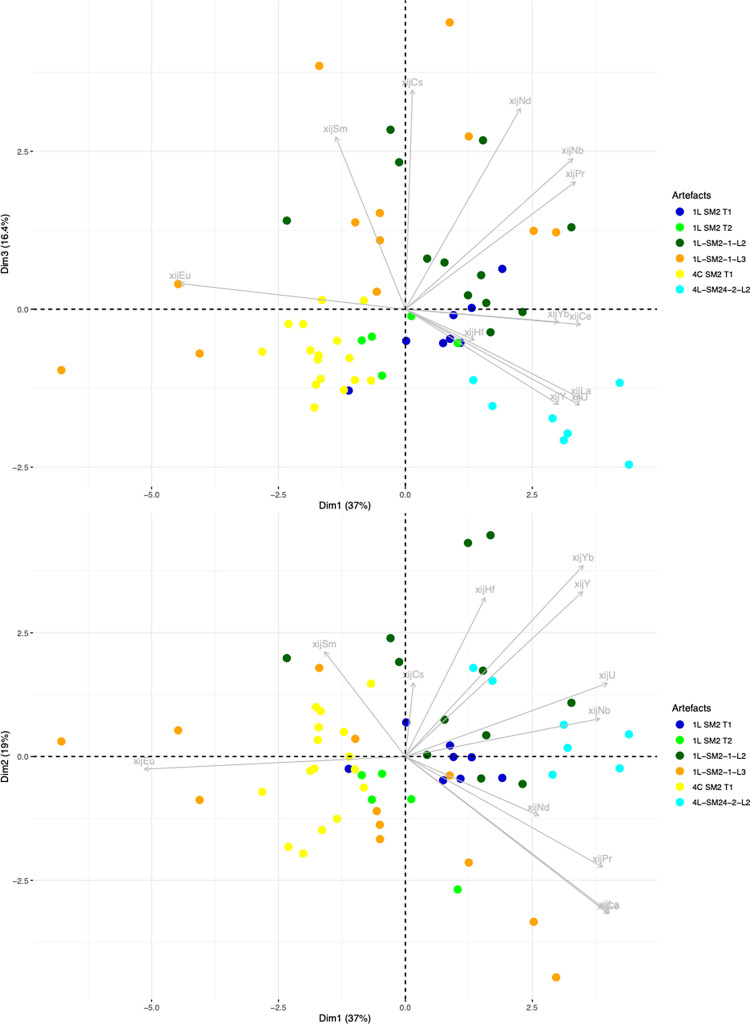
Stage 2 in statistical inference, PCA of the artefact group G1 (stage 1 of statistical inference). % of total variance.

To test the homogeneity of G1 group one step further, in a second stage of analysis, PCA was performed only on the bars/PPM belonging to this group. At this stage, PCA did not clearly distinguish among the bars in group G1 on the Dim1/Dim2/Dim3 projection (71% of the variance), except for 4L SM24-2-L2 (Fig 18) which seems to belong to a different group (G1a in Table 3).

**Fig 19:**
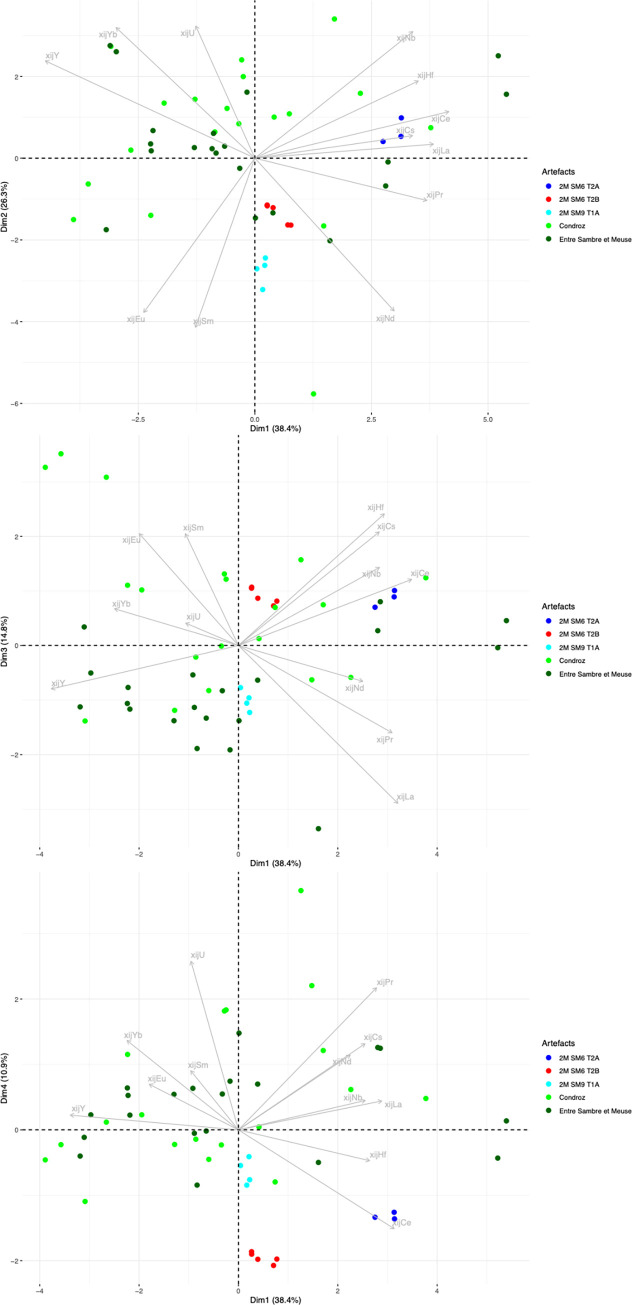
Stage 2 in statistical inference, PCA on the group of artefacts compatible with Wallonia from stage 1 of statistical inference. % of total variance.

**Fig 20:**
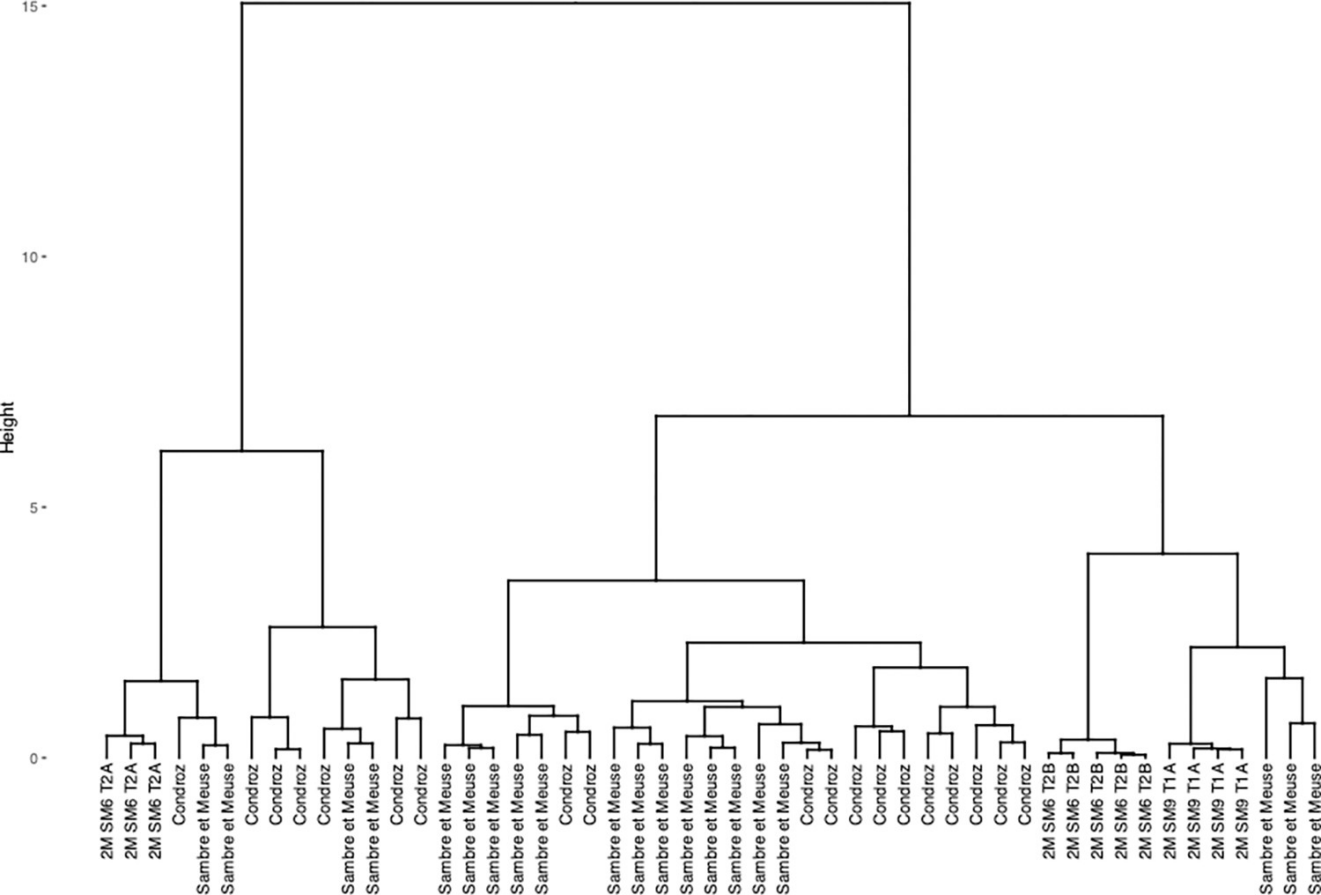
Dendrogram of the HAC analysis of Wallonia slag samples and artefacts 2M SM6 T2B and T2A.

The group of artefacts found to be compatible with a Wallonia production area during the first stage of the statistical inference (2M SM6 T2A and T2B and 2M SM9 T1A) was compared with Eastern Condroz and Entre-Sambre-et-Meuse only, using PCA. When Dim1, Dim2 and Dim3 (79% of the variance) were selected, the PCA did not distinguish any of the three artefacts from the production areas (Fig 19). When Dim4 was considered (90% of the variance), 2M SM6 T2A and B were slightly removed from the production area slag samples, although still close to the cluster. Nevertheless, the dendrogram resulting from a hierarchical agglomerative clustering (HAC) of this set of data does not place the PPM on separate branches from the Wallonia slag (Fig 20).

**Fig 21:**
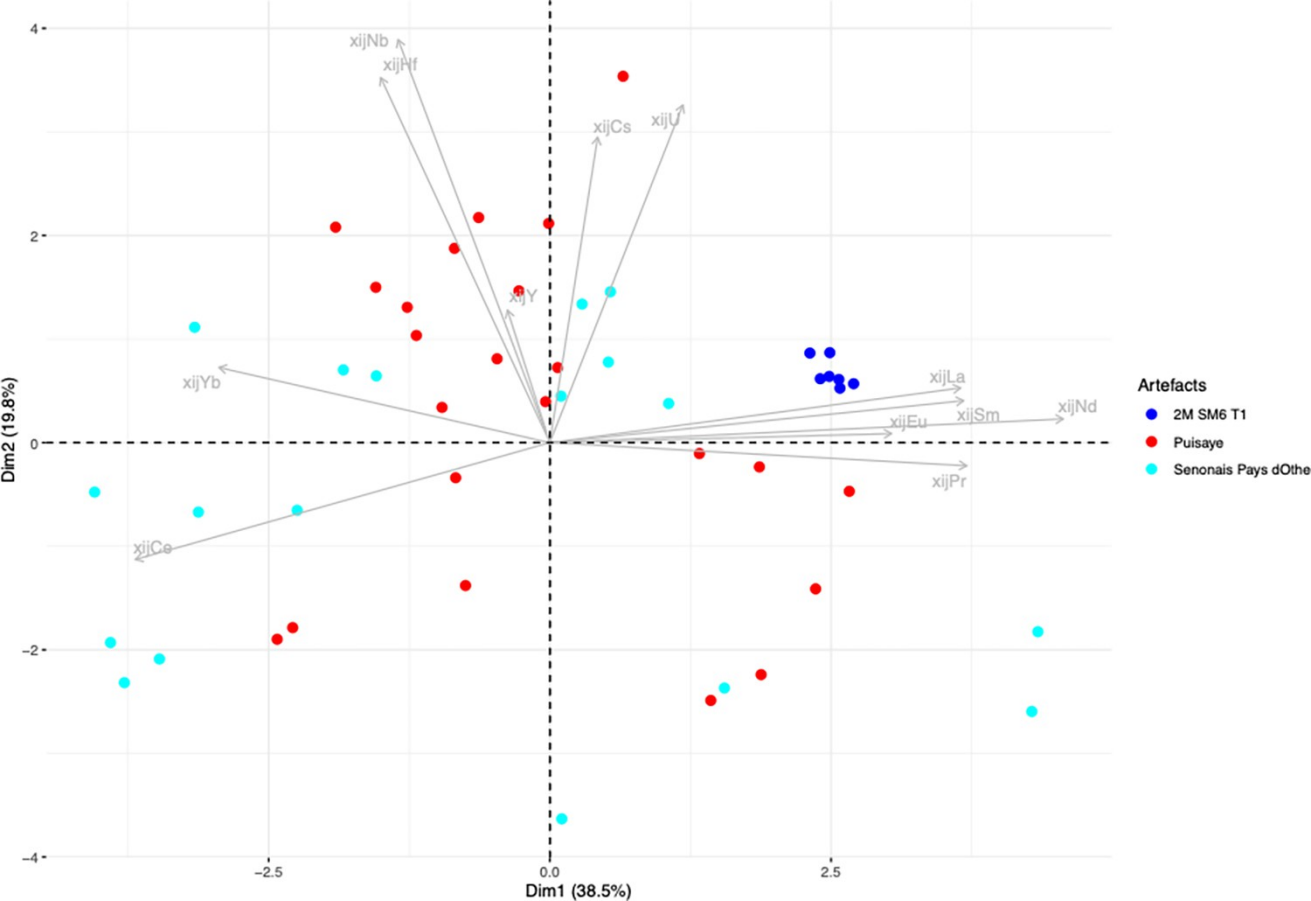
PCA of the artefact consistent with Sénonais-Pays-d’Hote/Puisaye during stage 1 of statistical inference. % of total variance.

When compared with only this production areas, the artefact consistent with Sénonais-Pays-d’Othe/Puisaye during the first stage of the inference (2M SM6 T1) is relatively distinct on the Dim1/Dim2 projection (Fig 21). Thus one may conclude that it belongs to a different provenance group (hereafter G5).

**Fig 22:**
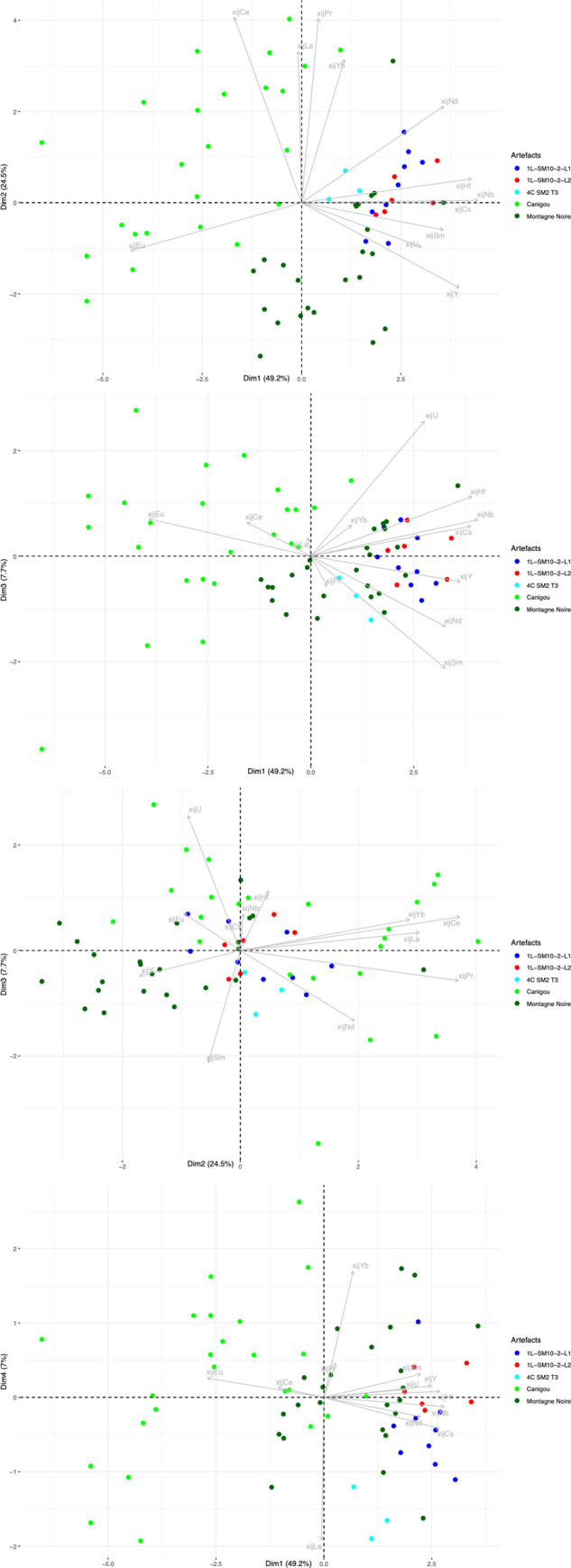
Stage 2 of statistical inference, PCA of the group of artefacts consistent with a Montagne Noire/Canigou provenance from stage 1 of statistical inference. % of total variance.

Lastly, the artefacts found to be consistent with the Montagne Noire/Canigou provenance (4C SM2 T3, 1L SM10 2 L1 and L2) during the first stage of statistical inference (Fig 17) are compared only with these two production areas. The results show that it is not possible to distinguish the slag inclusions of any of the artefacts from the cluster corresponding to slag from Montagne Noire using this approach, even by studying the projection of Dim1 to Dim4 (88.4% of the variance, Fig 22). It is also worth noting that none of the slag inclusions are consistent with slag from the Canigou area, only Montagne Noire. The same observations can be made by comparing the artefacts and the Montagne Noire area alone (results not shown).

To sum up these results, considering trace elements composition scaled and compared by PCA analysis and HAC and sometimes other complementary information (major elements composition), the studied PPM/artefacts can be put in different groups of provenances. Some of these groups are in good agreements with slag of considered production areas. All these results are showed in Table 3.

## 6. Discussion

Some of the bars studied have been attributed to a Montagne Noire origin, in previous studies based on a more limited number of chemical elements. The groups G2 and G3 from Coustures et al. [9] (1L SM2 T2/SM2-96-KL-Y2, 4C SM2 T1/SM2-4-B and 4C SM2 T3/ SM-2-3-A-1) were considered to be consistent with a Montagne Noire provenance. The present study, which analyses a wider spectrum of trace elements, clearly shows that our group G1 (corresponding to group G2 from Coustures et al. 2003) is not compatible with Montagne Noire. Among those bars analyzed by Coustures [9], only one (4C-SM2-T3) remains consistent with this origin. Nevertheless, in the present study, two new PPM appear to be compatible with the Montagne Noire production area: 1L SM10-2-L1 and L2. The major element content of the slag inclusions of these two PPM had already been studied by Pages [19]. Manganese was found only in the slag inclusions of the latter artefact (Table 2). This suggests that low levels of manganese in slag inclusions cannot be considered as an excluding factor for Montagne Noire, as confirmed by the variability of composition for this element in the slag.

In the present study, PPM 2L-SM9-T1A presents slag inclusions that are compatible with the chemical signature of slag from Wallonia (Eastern Condroz and Entre-Sambre-et-Meuse), as suggested by the principal component analysis. The case of the other two PPM (2M-SM6-T2A and T2B) must be discussed here in more detail. In the first stage of statistical inference (during which all chemical signatures were compared), the signature of the slag inclusions of these two PPM fit in with the Wallonia area on all the projections. Again, the signatures were consistent with Wallonia on projections for the first three components (79% of variance) when the two samples were compared to slag from Wallonia only. Unfortunately, once a fourth component was added (90% of variance), the slag inclusions of the two PPMs no longer plotted in the Wallonia areas in the Dim1/Dim4 projection. And yet, when hierarchical agglomerative clustering (HAC) was performed on the same set of data, the inclusions of the two PPM were not placed on a separate branch from the Wallonia slag. As a result, it can be considered that the chemical signatures of the inclusions are very close, although not identical, to the Wallonia slag. This is a situation where the decision to attribute an artefact to a given production area is not straightforward. The Wallonia production area is defined by a relatively low number of slag samples. Moreover, slight variations in reduction processes could result in chemical signature variations of the same order of magnitude as the difference between the SI composition of 2M-SM6-T2A and T2B. In light of these considerations, we have decided to attribute these two PPM to one of the Wallonia production areas from now on.

It is interesting to note that all the PPM with slag inclusions containing phosphorus– 4L-SM24-1-L1, L3, L4 and 4L SM24-2-L1 [19]–are located in the same area of the PCA projection when all PPM are considered. This suggests that these bars have the same provenance area, so this hypothesis will be considered to be true from now on. The SIs of 4L-SM24-2-L3 are also located in the same zone on the PCA projection, despite the fact that no P was detected in the slag inclusions [19].Therefore this PPM was considered to be part of a separate group of provenance: GP2.

**Fig 23:**
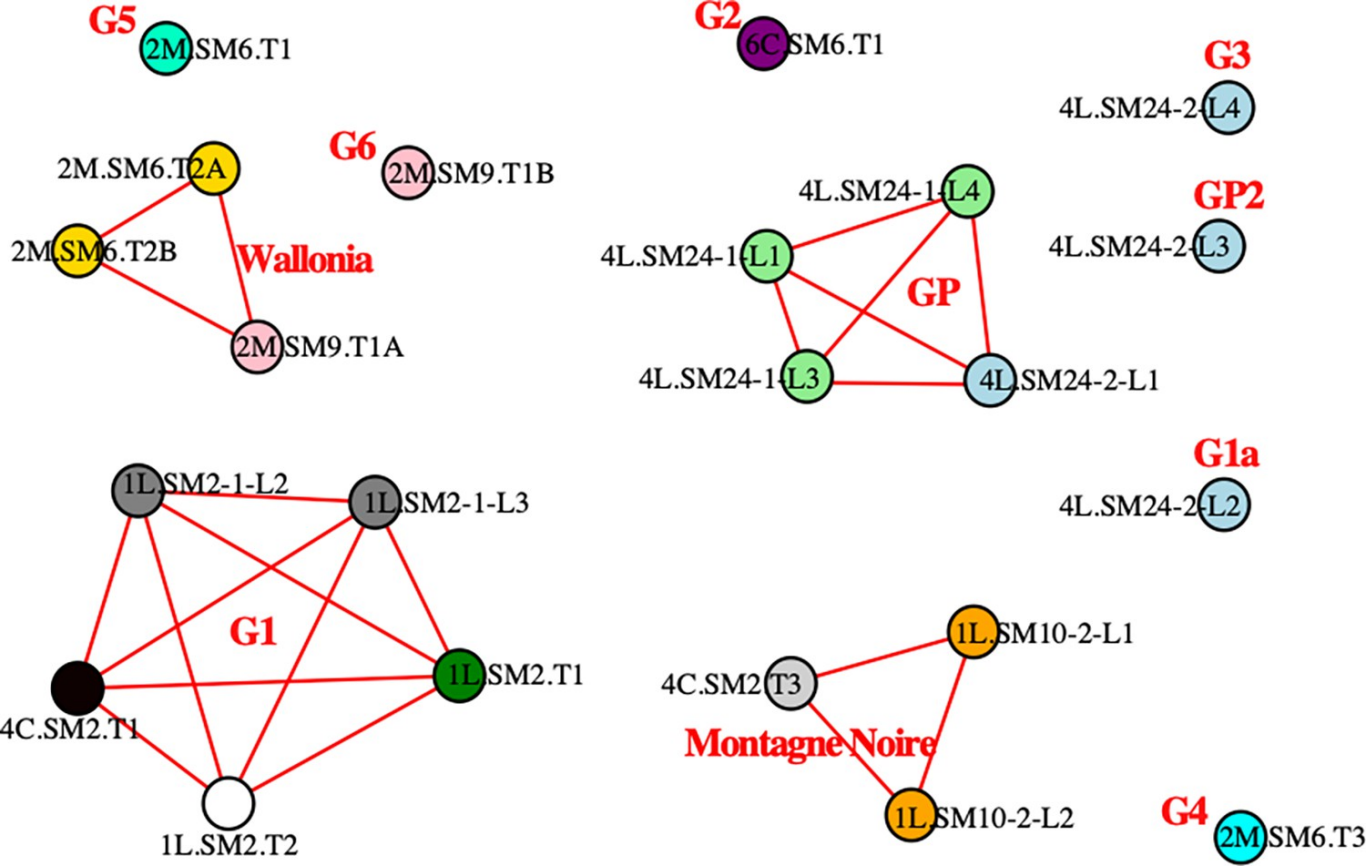
Network analysis: PPM from the same provenance are linked. PPM of the same color belong to the same bar. In red, the provenance network and name.

The highlighted similarities correspond to a chemical reality that we can take into account today to hypothesize about distribution networks in ancient times. Fig 23 shows the network built based on PPM provenance. Each PPM is a node that is linked to the other one if it belongs to the same provenance group. PPM coming from the same bar are shown in the same color. It displays a diversity of production origins, which reflects the abundance of smelting sites that existed in the western Roman Empire. Eleven different sources were found for the 22 PPMs analyzed in 13 bars. Some bars are made up of PPMs originating from the same source: this is the case for 2M SM6 T2, 1L SM10-2 and 1L SM2-1. Conversely, two bars were revealed to comprise multiple sources: these are 4L SM24-2 and 2M SM9 T1. Therefore, one bar can encompass multiple provenances if it is made of several PPMs. The manufacture of bars from iron of different origins has been evidenced for protohistoric and medieval semi-finished products and has long been assumed for the Roman period [19,53–55]. This proves that at least some bars were manufactured in specialized workshops separate from the smelting sites. Roman authors support this segmentation of production stages at separate sites. Diodorus of Sicily, a contemporary of the Saintes-Maries-de-la-Mer bars (Universal History, Book V, XIII), wrote that on the island of Elba, "*They melt the blocks [of ore] with the force of fire and share them to reach appropriate dimensions*, *and their appearance is similar to large sponges*. *Traders buy or exchange them and bring them to Dicaiarcheia [Pozzuoli] and other trading places*. *Some people buy this merchandise there and gather a large number of specialized blacksmiths who process it and make all kinds of iron figures out of it*.”

**Fig 24:**
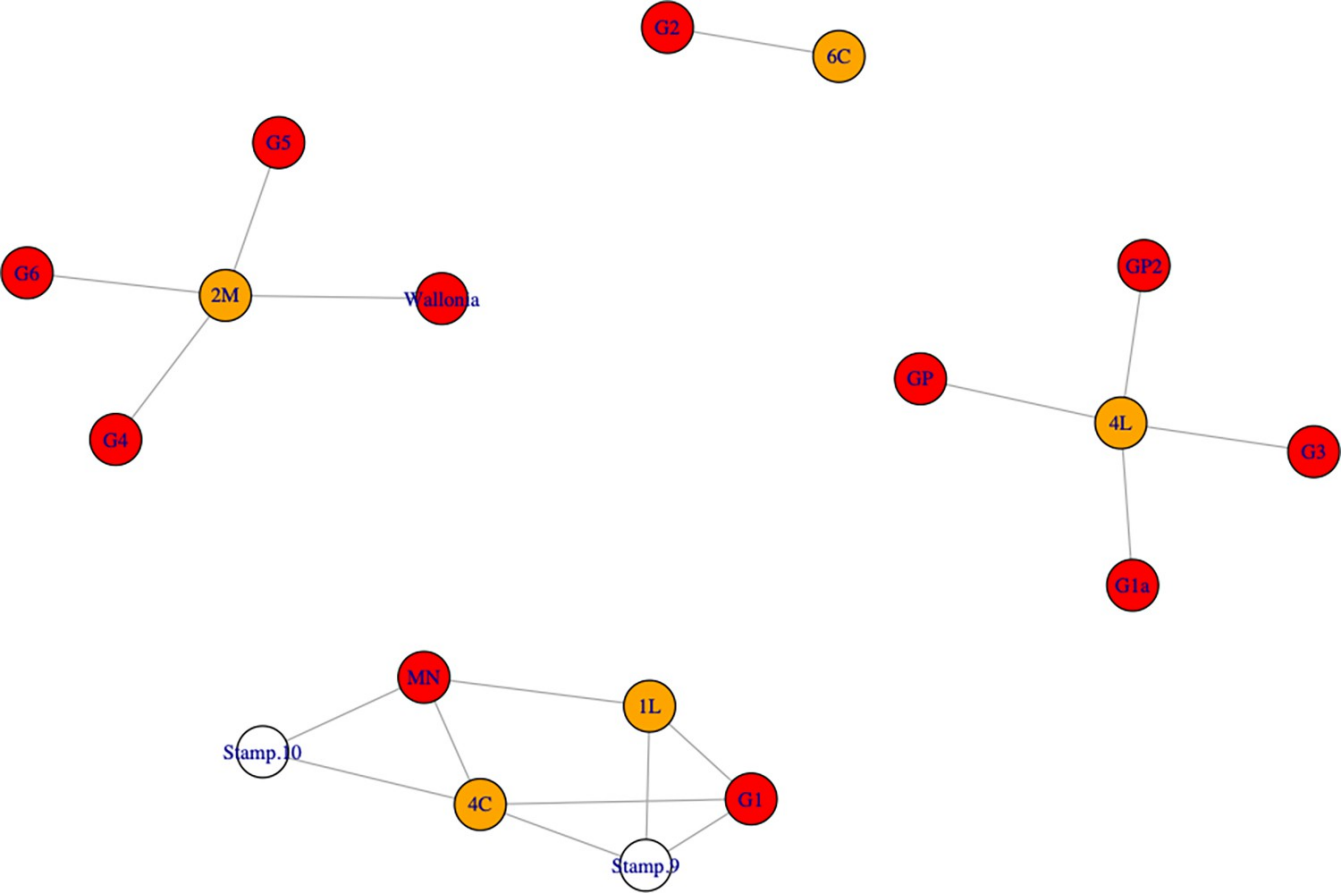
*Network analysis (connecting lines indicate the same provenance)*: P*rovenance (in red)*, *bar type (in orange) and stamp (in white)*. MN: Montagne Noire.

Another network analysis (Fig 24) can also be used to describe relationships between provenance groups and bar types (nodes). On the one hand, bar types 4L and 2M belong to specific provenance groups: four different sources for type 4L and four different sources for type 2M, including Wallonia. On the other hand, bar types 1L and 4C are both linked to two provenances, which are different from those for types 4L and 2M and include Montagne Noire. Type 6C is represented by one sample from yet another source (G2). And yet, previous studies have demonstrated that bar types are linked to iron quality [19]. Type 4L bars seems to be mainly made of phosphoric iron; 2M bars are ferrite and 1L and 4C are equivalent heterogeneous steel bars (categories defined by [19]). Type 4C bars have a short shape and made of mixed ferrite and steel. Type 1L bars have a long shape and also comprised of mixed ferrite and steel. This suggests that provenance groups are associated with specific iron qualities (or at leats types). The writings of Pliny the Elder (HN, liber XXXIV, XLI) confirm this view: “*This diversity [of irons] is due first of all to the nature of the soil and the climate; some lands provide only a soft*, *lead-like iron; others a brittle*, *coppery iron*, *which should not be used to make wheels and nails; the first species is suitable for these uses*.”

A given source is not associated with more than one quality of metal (for example, Wallonia is linked only to ferrite types; while G1 and Montagne Noire are also linked only to types made of mixed ferrite and steel). Likewise, each stamp is associated with only one provenance group: stamp 9 (*IVL//EROTIS*) with G1 and stamp 10 (*S//LEPEDI//N*) with Montagne Noire. However, a stamp may correspond to bars of different types with the same provenance. It should be noted that only three stamped bars were considered in our study. It is therefore difficult to draw conclusions about the significance of these stamps. As we have seen, we distinguish production areas defined by a same slag composition, not isolated smelting sites. Moreover bars are sometimes formed from different PPMs at specialist workshops that are distinct from the smelting sites. Therefore, it is possible that the iron bars were stamped in the workshops that assembled PPM to produce the bars.

**Fig 25:**
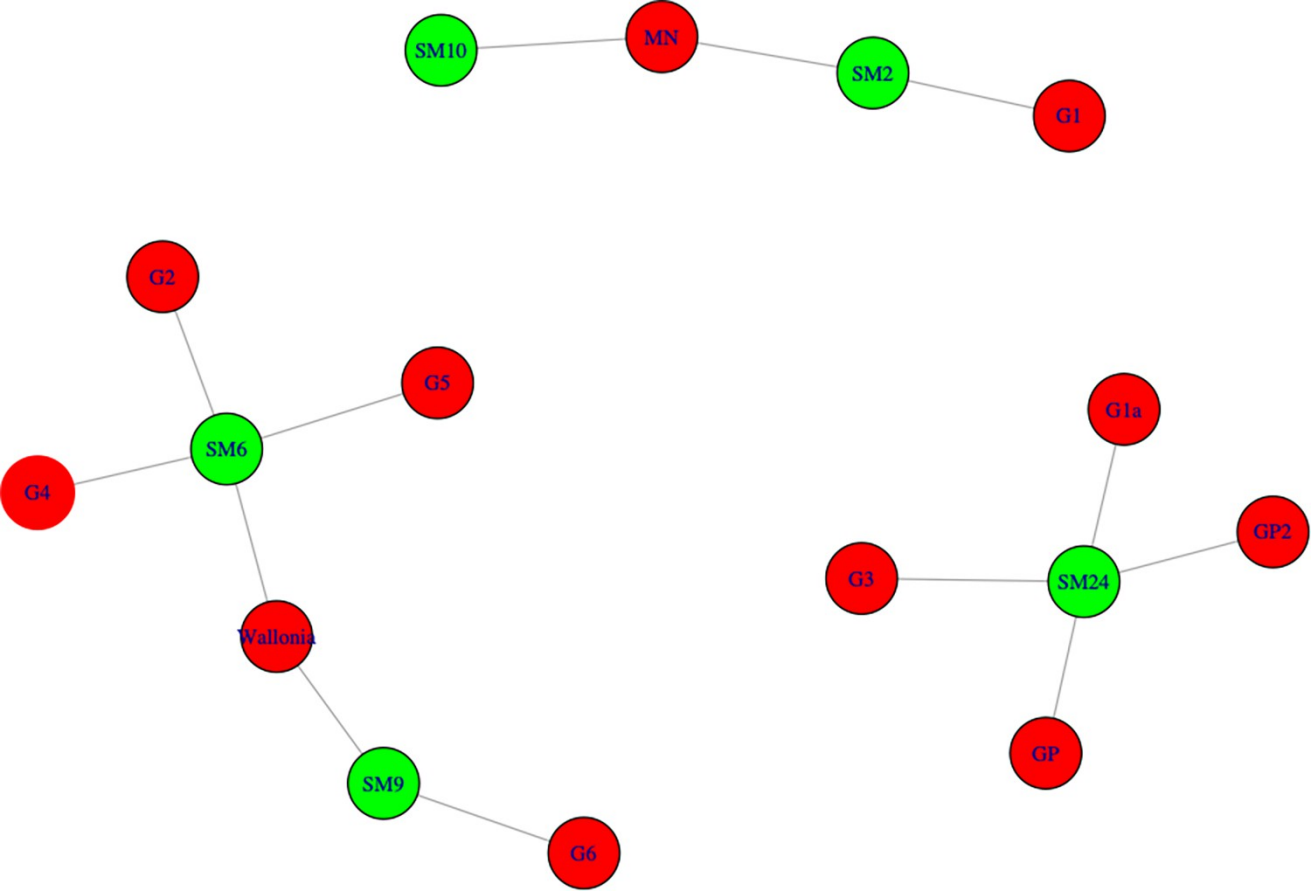
**Network analysis of shipwrecks (in green) and provenances (in red).** MN: Montagne Noire.

Next, if we plot a graph connecting shipwrecks and provenances of the bars constituting their loads (Fig 25), we see the formation of different clusters that do not contradict the other network analyses. It is interesting to note that the wrecks carried iron from different origins. However, the bars from Montagne Noire and Wallonia were not carried on the same ship. They apparently belong to two different trade networks: one for iron from the North of Gaul and the other for iron from the Mediterranean basin. The SM6 wreck assembles bars from Wallonia, as well as the unknown provenance groups G2, G4 and G5. These three groups are linked only to that wreck and bar types 2M and 6C. The SM9 wreck carried bars from Wallonia and from provenance group G6, which is not linked to any other shipwreck. It can therefore be assumed that these wrecks and the Wallonia provenance group belong to the same commercial network, with products coming from the North of Gaul. The SM10 and SM2 wrecks carried iron from Montagne Noire. The SM2 wreck also transported iron from the provenance group G1. Iron from this provenance group was found only in that wreck. Again, it would seem that these two wrecks and provenance groups form another coherent commercial network containing only 1L and 4C types of bars. Lastly, the SM24 wreck transported phosphoric iron (type 4L) from provenance GP as well as three other provenance groups (GP2, G1a and G3), very probably forming a third commercial network for iron from an unknown provenance.

**Fig 26:**
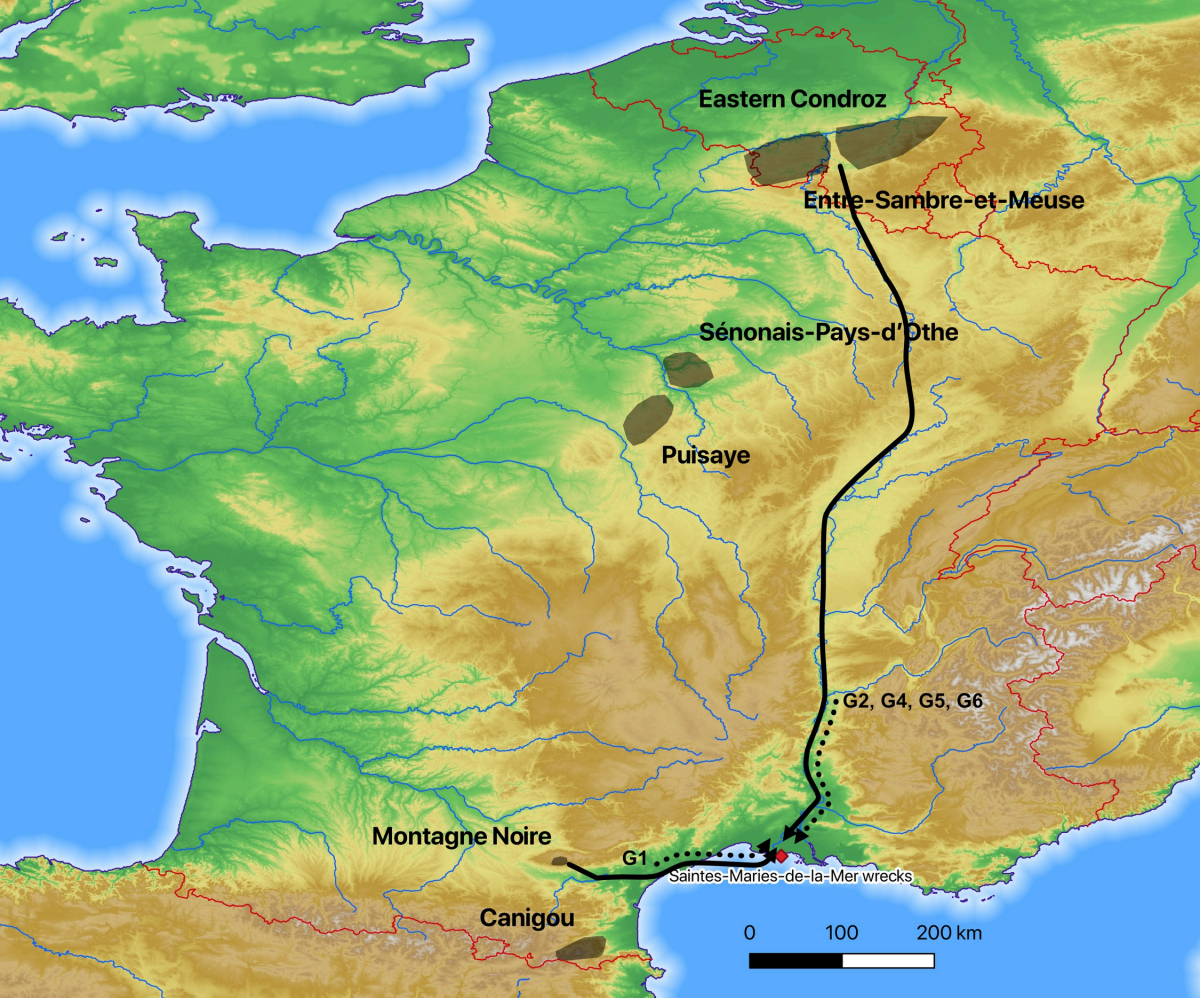
Direction and route of traffic from identified (solid line) and uncertain (dashed black line) provenance groups; as we have no identified provenance for wreck SM24, groups GP, G1a and G3 are not mentioned on this map (north at the top; DEM NASA Shuttle Radar Topography Mission Global 1 arc second).

Consequently, at least two different iron bar trade networks passed through the mouth of the Rhône River: one from northern Gaul that included Wallonia and one from the Mediterranean coast, probably near the Narbonne harbor, that included iron produced at Montagne Noire (Fig 26).

**Fig 27:**
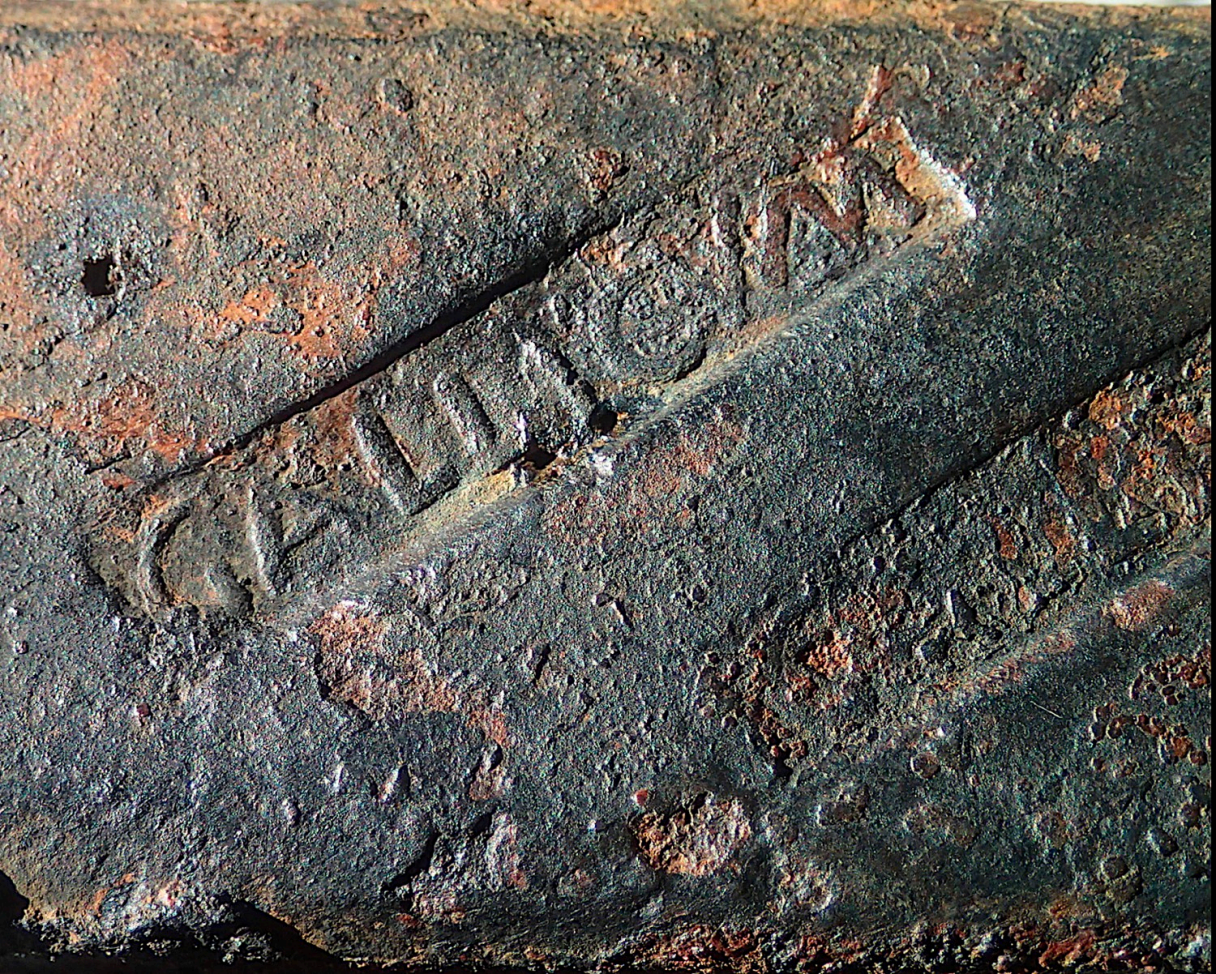
GALLICUM stamp discovered by Luc Long on a 2M-type bar from the SM33 wreck off Saintes-Maries-de-la-Mer.

Iron trade from northern Gaul via the Rhône to the Mediterranean dates back to at least the 2nd century BC and most likely the beginning or even before the Roman conquest of Gaul. Indeed, a set of 15 radiocarbon dates for two SM6 bars and two SM9 bars indicate that the transported iron was produced between 240 and 51 BC and between 196 and 69 BC, respectively [42]. The Bagaud 2 wreck (Hyères, Var, France, end of the 2nd century BC to the beginning of the 1st century BC) provides another example of this iron trade between Gaul to the Mediterranean prior to the Roman conquest [56–58]. This wreck carried tin ingots stamped *Hypokeltoi* (meaning "Celts from below" or "from the south") and type 2M and 4C iron bars. This metal trade from provinces close to Germania or from Germania is also attested between the 1st century BC and the 1st century AD by the SM1 wreck (Saintes-Maries-de-la-Mer, Bouches-du-Rhône, France) and other Mediterranean discoveries of lead ingot [58–61]. North-to-south iron trade via the Rhône continued until at least the middle of the 2nd century AD, based on data from the Saint-Gervais I wreck (Fos-sur-Mer, Bouches-du-Rhône, France), which contained lead ingots from *Britannia* and type 2M iron bars [58,62]. Stamps on iron bars also bear witness to this north-south trade in the ancient period, notably with the Aedui Gallic tribe (Bourgogne, France, type 2M) and Bituriges Cubes (Berry, France, type 1M) known for their metal production [63,64]. Also attesting to this trade is the recent discovery of a *GALLICUM* stamp by Luc Long on a type-2M bar from the SM33 wreck off Saintes-Maries-de-la-Mer (Fig 27). This trade developed along commercial routes already in use since the Bronze Age and through Protohistory for the trade of tin and iron, especially from the south of England [2,58,65]. It can be understood that this north-to-south trade was long anchored in the multiple iron production areas of Gaul, Germania and Britannia, as can be seen from the diversity of the chemical signatures (at least four) analyzed in this study.

The iron trade from the Mediterranean Sea via the Rhône to the North (at least as far as the Arles harbor) is partly linked to the ironmaking aicrea of Montagne Noire and therefore to the *Colonia Narbo Martius* (Narbonne, Aude France), the capital city of a Roman province whose harbor was, according to Strabo (IV, 1, 12), the “*main port of the whole of Gallia Celtica*” at the end of the 1st century AD. This trade appears to be more recent than that developed in the north after the conquest of Gaul. The SM2 and SM10 ships would have circulated between 23 BC and 60 AD and between 5 and 163 AD, respectively, according to the 15 radiocarbon dates for four bars [42]. No further evidence of south-to-north iron trade via the Rhône is provided by archaeological, archaeometric or epigraphic documentation. Moreover, generally speaking, the trade in metals from south to north seems much less significant than from north to south [58]: only two sources refer to this area. The reason for this imbalance is that the production of metals (especially iron) is greater in the north of the Empire than around the western Mediterranean basin [5,66]. During the Roman Empire, metals could be found only around Elba island and Populonia or around Narbonne, in the Montagne Noire area and the Canigou massif, and to a lesser extent in Corbière and Ariège.

## Conclusion

This study focused on the trace element analysis of slag inclusions embedded in bars from the largest known set of Roman wrecks carrying cargoes of iron: the Saintes-Maries-de-la-Mer wrecks. The results were compared to the composition of slag from the six largest iron-producing areas in the western Roman Empire. A statistical approach, mainly using PCA, enabled us to link some bars with multiple provenance groups, which did or did not include the production areas being analyzed. Our interpretations are based on current data, resulting from 20 years of research. Although these analyses will need to be completed in the future, they already offer a glimpse of what appear to be different networks linking places of production to places of consumption.

Indeed, at the end of the Iron Age and during the Roman conquest of Hispania and Gaul, iron became a common, everyday material used extensively in all aspects of Gallo-Roman life and found in all categories of material culture. This large-scale, universal use of iron was fueled by the development of large iron production areas, especially in Gaul, and on intensive, crisscrossing trade and distribution flows. Being a heavy material, large quantities of iron were transported by ship over long distances via the Mediterranean Sea and the rivers flowing into it. A complex system of intertwining commercial routes supplied iron to the whole of the expanding Roman Empire. Thanks to our study, we can see that iron from some regions, including Wallonia (Belgium), was carried down the Rhône, while iron from other regions, including Montagne Noire (France), was carried up the river. The intricacy of this sales and distribution network can be partly explained by the fact that each ironmaking area seems to have been more likely to produce a particular quality of iron. Iron is in fact a metal with multiple qualities, of which ferrite, steel, and phosphoric iron are just a few. The ancient iron economy is therefore an important sector of the world economy that merits further study.

## Supporting information

S1 Table Chemical data of iron production areas and slag inclusions of Primary Pieces of Metal (PPM) of iron bars. (XLSX)
