# Correction: Vice-versa: The iron trade in the western Roman Empire between Gaul and the Mediterranean

**DOI:** 10.1371/journal.pone.0310030

**Published:** 2024-09-03

**Authors:** Gaspard Pagès, Philippe Dillmann, Enrique Vega, Marion Berranger, Sylvain Bauvais, Luc Long, Philippe Fluzin

There is an error in affiliation 2 for authors Philippe Dillmann, Enrique Vega and Sylvain Bauvais. The correct affiliation 2 is: CNRS, LAPA-IRAMAT UMR7065, NIMBE UMR3685, CEA, CNRS, Université Paris-Saclay, CEA Saclay 91191, Gif-sur-Yvette, France.

There are errors in the Author Contributions. The correct contributions are:

**Conceptualization:** Gaspard Pagès, Philippe Dillmann

**Writing–original draft:** Gaspard Pagès, Philippe Dillmann

**Data Acquisition:** Enrique Vega, Marion Berranger, Sylvain Bauvais, Luc Long

**Data Processing:** Enrique Vega, Philippe Fluzin

The ORCID iDs are missing for the second and fourth author. Please see the authors’ respective ORCID iDs here:

- Author Philippe Dillmann ORCID iD is: 0000-0003-3226-1611 (https://orcid.org/0000-0003-3226-1611).
- Author Marion Berranger ORCID iD is: 0000-0002-9454-2913 (https://orcid.org/0000-0002-9454-2913).

## References

[1] Pagès G, Dillmann P, Vega E, Berranger M, Bauvais S, Long L, et al. (2022) Vice-versa: The iron trade in the western Roman Empire between Gaul and the Mediterranean. PLOS ONE 17(5): e0268209. 10.1371/journal.pone.0268209 35580132 PMC9113607

